# Differential Expression Analysis of Single-Cell RNA-Seq Data: Current Statistical Approaches and Outstanding Challenges

**DOI:** 10.3390/e24070995

**Published:** 2022-07-18

**Authors:** Samarendra Das, Anil Rai, Shesh N. Rai

**Affiliations:** 1 ICAR-Directorate of Foot and Mouth Disease, Arugul, Bhubaneswar 752050, India; 2 International Centre for Foot and Mouth Disease, Arugul, Bhubaneswar 752050, India; 3 ICAR-Indian Agricultural Statistics Research Institute, PUSA, New Delhi 110012, India; anil.rai@icar.gov.in; 4 School of Interdisciplinary and Graduate Studies, University of Louisville, Louisville, KY 40292, USA; 5 Biostatistics and Bioinformatics Facility, Brown Cancer Center, University of Louisville, Louisville, KY 40202, USA; 6 Biostatisitcs and Informatics Facility, Center for Integrative Environmental Health Sciences, University of Louisville, Louisville, KY 40202, USA; 7 Data Analysis and Sample Management Facility, The University of Louisville Super Fund Center, University of Louisville, Louisville, KY 40202, USA; 8 Hepatobiology and Toxicology Center, University of Louisville, Louisville, KY 40202, USA; 9 Christina Lee Brown Envirome Institute, University of Louisville, Louisville, KY 40202, USA

**Keywords:** scRNA-seq, differential expression analysis, classification, statistical approaches, challenges

## Abstract

With the advent of single-cell RNA-sequencing (scRNA-seq), it is possible to measure the expression dynamics of genes at the single-cell level. Through scRNA-seq, a huge amount of expression data for several thousand(s) of genes over million(s) of cells are generated in a single experiment. Differential expression analysis is the primary downstream analysis of such data to identify gene markers for cell type detection and also provide inputs to other secondary analyses. Many statistical approaches for differential expression analysis have been reported in the literature. Therefore, we critically discuss the underlying statistical principles of the approaches and distinctly divide them into six major classes, i.e., generalized linear, generalized additive, Hurdle, mixture models, two-class parametric, and non-parametric approaches. We also succinctly discuss the limitations that are specific to each class of approaches, and how they are addressed by other subsequent classes of approach. A number of challenges are identified in this study that must be addressed to develop the next class of innovative approaches. Furthermore, we also emphasize the methodological challenges involved in differential expression analysis of scRNA-seq data that researchers must address to draw maximum benefit from this recent single-cell technology. This study will serve as a guide to genome researchers and experimental biologists to objectively select options for their analysis.

## 1. Background

High-throughput single-cell RNA-sequencing (scRNA-seq) has emerged as a promising technology to explore the dynamics of gene expression at the single-cell level. It has become extremely popular for answering the key questions of developmental biology, including cellular heterogeneity study [1], the discovery of novel cell types [2], and cell trajectory analysis [3], etc. To date, many single-cell sequencing protocols have been developed, of which two are very popular: (i) unique molecular identifier (UMI) tag-based protocols such as Drop-seq [4] and 10x Genomics Chromium [5]; and, (ii) full length, non-UMI-based protocols, e.g., Smart-seq2 and Fluidigm C1 [6,7]. UMI-based protocols sequence only the 5-prime or 3-prime end of the mRNA molecule compared with non-UMI protocols [8]. The former has lesser amplification bias (i.e., transcript isoforms within the same gene) compared with the latter [9]. Irrespective of the sequencing protocols, scRNA-seq data have some peculiar features including high-level noises, excess overdispersion, low library sizes, sparsity, and a higher proportion of zeros (i.e., due to the lower capture of transcriptomic material and other sources of variation), etc., [10]. Through these single-cell protocols, a huge amount of gene expression data (over thousand(s) to millions of cells) are generated in each experiment and deposited in public domain databases. Such an unprecedented event requires novel and advanced statistical approaches and bioinformatics tools to extract relevant biological knowledge.

Differential expression analysis (DEA) is the primary downstream analysis performed on scRNA-seq data [11,12,13]. The DEA is useful for the detection of biomarkers for novel cell types or gene signatures for cellular heterogeneity, and also provides inputs for other secondary analyses including gene set or pathway, and network analysis. The initial practice of DEA in scRNA-seq involved borrowing methods from bulk RNA-seq, which usually did not consider the special features of the scRNA-seq data [10,14]. Hence, specialized approaches have been reported in the literature for DEA of scRNA-seq data. Software(s)/R packages were developed based on these statistical approaches. Each approach has its own benefits and drawbacks, i.e., DEA approaches have distinct features and disparate performances. Several computational experiments have been conducted to establish the same, as reported in the literature [10,11,14,15,16,17,18]. Excellent review(s) of the computational comparative studies can be found in [10,16]. However, the major chunk of the assessed approaches was imported from the bulk RNA-seq. For instance, Soneson and Robinson (2019) and Das et al. (2021) considered ~50% approaches from the bulk RNA-seq to assess the DEA approaches’ performance on scRNA-seq datasets [10,16]. There are limited studies available in the literature which mainly focuses on critically reviewing DEA approaches exclusively designed for single-cell studies.

Therefore, in this review, we aim to present: (i) state-of-the-art methods and tools available for DEA of scRNA-seq data along with their classification based on input data, fitted statistical models, and test statistic(s); (ii) discuss the unique features and limitations of each class of approaches; and (iii) describe the key challenges yet to be addressed in the DEA of the scRNA-seq data. This study will serve as a catalog and provide guidelines to genome researchers and experimental biologists for objectively choosing proper DEA approaches, based on several factors.

## 2. Current Statistical Approaches

**Figure 1:**
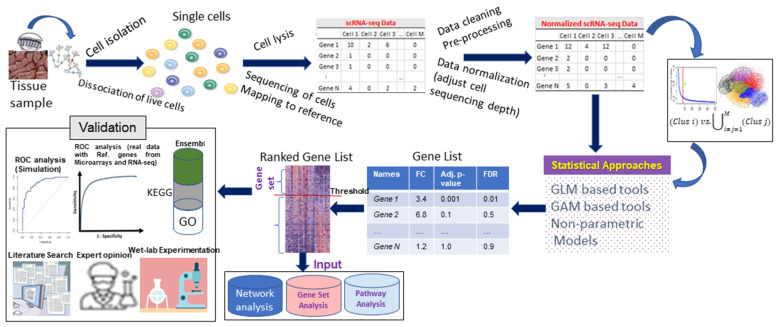
**Operational framework of differential expression analysis of scRNA-seq data.** Various steps in single-cell studies are shown. Pre-processing and various steps of DE analysis are also shown. Potential use and interpretation of obtained results are presented.

The term “Differential Expression” has been extensively used in gene expression studies including microarrays [19], RNA-seq [20], and scRNA-seq [16]. The basic difference of DEA for scRNA-seq, compared with other studies, is that it is used to detect bio-markers across the cell types, while in other studies it is used to find differential genes across the case vs. control conditions [21,22]. The operational framework of the DEA in scRNA-seq study is shown in Figure 1. The operational procedure is mostly common to single-cell studies (Figure 1). It is beyond the scope of this article to discuss the large number of existing analytical approaches covered by the term ‘Differential Expression Analysis’ in gene expression studies. Therefore, this review only focuses on statistical approaches that were exclusively developed for single-cell expression studies, rather than on methods that perform DEA on any sequencing data.

**Figure 2:**
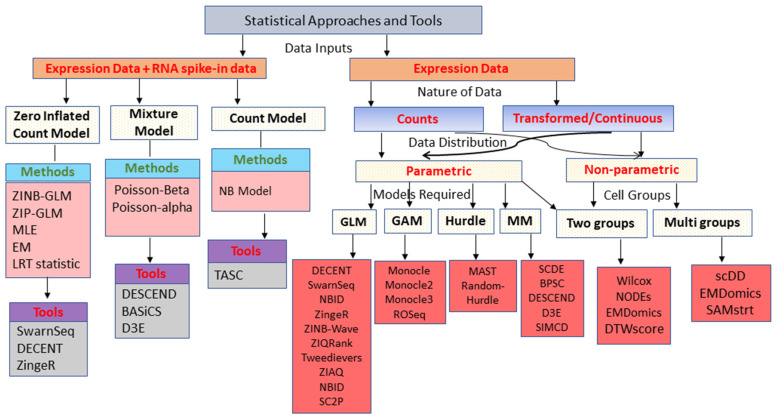
**Classification of available statistical approaches and tools used for DEA in single-cell studies.** Classification of the approaches is conducted based on the requirement of input data, data distribution, and statistical models, etc. DE analytic tools belonging to each category are presented in pink colored boxes.

The existing approaches and tools for the DEA of scRNA-seq data and their availability are listed in Table 1 and Table S3. Table 1 also presents a comparative overview of the approaches in terms of different factors including statistical models, input, test statistic(s), and runtime, etc. We also classified these existing approaches and tools based on several factors including data requirement and background models, etc., as shown in Figure 2. For instance, the available approaches can be grouped based on the input data requirement, i.e., (i) group of approaches, which requires expression data as well as external spike-in data; (ii) other group of approaches, which only requires expression data (Figure 2). Further, the former category of tools is computationally expensive and more accurate compared with the latter [23], due to the implementation of efficient statistical models and spike-ins data requirement. The use of RNA spike-ins in the approaches tackles the issue of technical variability due to inefficient transcriptomes capture in single-cells [24,25,26,27,28]. In other words, the RNA spike-ins are RNA transcripts (with known sequences and quantity) that are applied to calibrate the measurements of RNA hybridization assays, such that scRNA-Seq and UMIs can theoretically enable the estimation of absolute molecular counts [29]. It is worthy to note that, if RNA spike-ins data are available, it is profitable to use them in DEA, using a suitable approach. Moreover, the classification of the approaches and tools based on other factors can be found in Figure 2.

Instead of individually reviewing the large number of DEA approaches (Table 1), our goal here is to classify the approaches based on the common statistical principles/models and discuss their relative merits. However, for the researcher desiring specific information about the individual tools, Supplementary Document S1 briefly presents reviews of the individual approaches. We also present class-wise critical reviews of the existing DEA approaches in the following section.

## 3. Classes of Statistical Approaches for DEA

***Notation*:**$Y_{ij}$: random variable (*rv*) represents the observed expression (i.e., read, UMI) counts of *i*th (*i* = 1, 2, …, *N*) gene in *j*th (*j* = 1, 2, …, *M*) cell; *N*: total number of genes; *M*: total number of cells; $\mu_{i}$: mean of *i*th gene for NB distribution (count part of the model); $\varphi_{i}$ and $\theta_{i}$ $\left(=\varphi_{i}^{-1}\right)$: dispersion and size parameters, respectively, for *i*th gene; $\pi_{i}\left(\in \left[0,\;1\right]\right)$: mixture probability (zero inflation probability) of *i*th gene; $s_{j}$: library size of *j*th cell; $Z_{ij}$: *rv* represents the true (unknown) expression counts for *i*th gene in *j*th cell; X: design matrix for cell group information, whose *j*th row: $X_{j}=\left[X_{j1},\;X_{j2},\ldots ,\;X_{jN}\right]$; $W_{ij}$: indicator *rv* representing the rate of expression for *i*th gene in *j*th cell, i.e., $W_{ij}=0:\;Y_{ij}=0;\;W_{ij}=1:\;Y_{ij}>0$. $\Omega_{i}=\left\{\mu_{i},\;\theta_{i},\pi_{i}\right\}$: parametric space for *i*th gene.

### 3.1. Generalized Linear Model-Based Approaches

**Figure 3:**
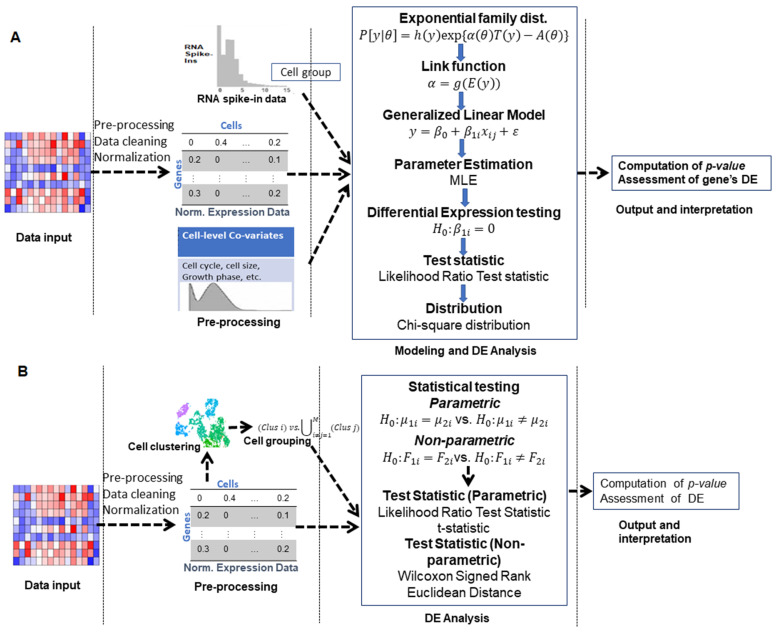
**Operational outlines of DE analytic GLM and two-class comparison approaches in scRNA-seq studies.** (**A**) Workflow of steps for GLM-based DE approaches. (**B**) Workflow of steps for two-class comparison approaches. In both classes, the framework can be divided into four major parts, namely: (i) input (data provided as input to tools); (ii) pre-processing of data, this step involves data cleaning, outlier removal, normalization, etc.; (iii) model fitting and computation of DE test statistic, various distributional/model (e.g., GLM, simple statistical distribution or distribution-free) assumptions are made about the expression data, parameters of the models are estimated, and DE test statistic(s) for genes and their corresponding p-values are computed; and, (iv) assessment and interpretation of DE results.

Generalized linear model (GLM)-based approaches assume that: (i) expression counts follow certain exponential family distribution [62]; and, (ii) a non-linear function (known as *link function*) relates the expected expression counts of genes to the linear component of the model. In other words, every GLM has three components: (i) expression count distribution of the gene (sometimes called the error structure); (ii) linear predictor that involves the explanatory cell variables or covariates including cell group information; and, (iii) a link function (g(.)) that connects the linear predictor to the natural mean of expression counts of genes. The GLM class of DEA approaches includes NBID [30], DECENT [24], ZINB–WaVE [31], ZingeR [32,33], Tweedieverse [34], and SwarnSeq [13], to name a few. The operational layout of this class of approaches is shown in Figure 3.

The GLM-based DEA approaches can be divided into two categories: first, expression counts follow certain exponential family distributions, e.g., negative binomial (NB) and Poisson, etc. Second, expression counts follow zero-inflated models, e.g., zero inflated negative binomial (ZINB), and zero inflated Poisson (ZIP) (Supplementary Document S2). For instance, NBID [30] and IDEAS [53] approaches use the NB model, and probability mass function (PMF) in Equation (1), to fit the single-cell expression counts data. The expected value and variance of the observed expression counts is given in Equation (2).

(1) $$P\left[Y_{ij}=y\right]=\frac{G\left(y+\theta_{ij}\right)}{G\left(y+1\right)G\left(\theta_{ij}\right)}{\left(\frac{\theta_{ij}}{\theta_{ij}+\mu_{ij}}\right)}^{\theta_{ij}}{\left(\frac{\mu_{ij}}{\theta_{ij}+\mu_{ij}}\right)}^{y}\;\forall \;y=0,\;1,\;2,\;\ldots$$

where, $\mu_{ij}\geq 0;\;\theta_{ij}>0$ are the mean and size parameters of NB distribution, *G*(.): Gamma function. Then, the expected value and variance of $Y_{ij}$ is shown as:

(2) $$E\left(Y_{ij}\right)=\mu_{ij}\;\mathrm{and}\;V\left(Y_{ij}\right)=\mu_{ij}+\frac{\mu_{ij}{}^{2}}{\theta_{ij}}=\mu_{ij}+\varphi_{ij}$$

The NBID uses a non-linear link function to model the expected value of expression counts with the explanatory variables, such as cell group labels and other potential covariates, given in Equation (3).

(3) $$g\left(\mu_{ij}\right)=log\mu_{ij}=X\beta$$

where, g(.): link function, and β: parameters of the model.

The NB–GLM approaches may not suitable to fit the scRNA-seq counts data due to the presence of excess zeros (Supplementary Documents S3–S5) [10,13,32], thus may compromise the statistical power to detect true differentially expressed genes [13,31]. Hence, ZIM was introduced in DEA bioinformatics tools to fit the observed scRNA-seq count data [1,2,3,4,5,6,7,8,9,10,11,12,13,14,15,16,17,18,19,20,21,22,23,24,25,26,27,28,29,30,31,32,33]. The ZIM–GLM-based approaches include tools such as DECENT [24], ZINB–WaVE [31], ZingeR [32,33], and SwarnSeq [13], which assume the UMI counts of genes follow a ZINB distribution. PMF is given in Equation (4).

(4) $$P\left[Y_{ij}=y\right]=\{\begin{array}{c} \pi_{ij}+\left(1-\pi_{ij}\right){\left(\frac{\theta_{ij}}{\theta_{ij}+\mu_{ij}}\right)}^{\theta_{ij}}\;when\;y=0\\ \left(1-\pi_{ij}\right)\frac{G\left(y+\theta_{ij}\right)}{G\left(y+1\right)G\left(\theta_{ij}\right)}{\left(\frac{\theta_{ij}}{\theta_{ij}+\mu_{ij}}\right)}^{\theta_{ij}}{\left(\frac{\mu_{ij}}{\theta_{ij}+\mu_{ij}}\right)}^{y};\;y>0 \end{array}$$

The expected value and variance of $Y_{ij}$ is expressed in Equations (5) and (6).

(5) $$E\left(Y_{ij}\right)=\left(1-\pi_{ij}\right)\mu_{ij}$$

(6) $$V\left(Y_{ij}\right)=\left(1-\pi_{ij}\right)\mu_{ij}\left(1+\pi_{ij}\mu_{ij}+\frac{\mu_{ij}}{\theta_{ij}}\right)$$

If $\pi_{ij}=0$ $\dot{\Rightarrow }$ $ZINB\left(\pi_{ij},\mu_{ij},\theta_{ij}\right)\to NB\left(\mu_{ij},\theta_{ij}\right)$

If $\varphi_{ij}\to 0\dot{\Rightarrow }\;ZINB\left(\pi_{ij},\mu_{ij},\theta_{ij}\right)\;\to ZIP\left(\pi_{ij},\mu_{ij}\right)$

The linear predictors including cellular group [24], cell type [13], and other cell-level auxiliaries [31] are included in the GLM. Various link functions including log and logit functions are used to model the gene specific mean and zero-inflation parameters, respectively. For instance, DECENT, SwarnSeq, ZINB–Wave, and ZingeR consider log-link function to connect the mean with cell group, cell-level auxiliary predictors and *logit*-link function, to model the zero-inflation parameter, as given in Equations (7) and (8).

(7) $$log\mu_{i}=X\gamma_{i}+Rw_{i}+Cs_{i}+O_{\mu }$$

(8) $$logit\pi_{i}=X\beta_{i}+Ru_{i}+Cv_{i}+O_{\pi }$$

where, $logit\left(\pi \right)=log\left(\frac{\pi }{1-\pi }\right)$; $\alpha_{i}$, $\tau_{i}$ and $\varphi_{i}$**:** *M* × 1 vector of parameters for *i*th gene; ***X*:** *M* × *G* design matrix providing group information (first column consists of 1 s to include intercept term); *G*: number of cellular groups (cell clusters are divided into *G* groups, if group is unknown); ***R***: *M* × *N* design matrix providing cell cluster information; ***C***: *M* × *C* design matrix providing cell level auxiliary information; $\gamma_{i}$ and $\beta_{i}$**:** *G* × *1* vectors of cellular groups effects for *i*th gene; $w_{i}$ and $u_{i}$: *N* × *1* vectors of cell cluster effects for *i*th gene; $s_{i}$ and $v_{i}$: *C* × *1* vectors of effects for cell level co-variates, such as cell cycle, cell phase, etc., for the *i*th gene; *C*: levels of cell level auxiliaries; and, $O_{\mu },\;O_{\pi }$: offsets for $\mu_{i}$ and $\pi_{k}$, respectively.

The DECENT approach only considers the cell group information as predictor in the model (Equation (9)), while the SwarnSeq, ZingeR and ZINB–WaVE approaches consider group as well as other cell level data as linear predictors in the model (Equations (7) and (8)). All GLM-based approaches use the maximum likelihood estimation (MLE) method to estimate the model parameters through minimizing the goodness of fit criterion [63]. However, it is very difficult to obtain the exact solution (i.e., closed form) of the MLE’s objective function [13,33]. Therefore, iterative expected maximization (EM) techniques are implemented in these approaches to estimate the parameters [13,32]. For instance, DECENT, ZingeR, ZINB–WaVE, and SwarnSeq use EM algorithms to estimate the gene specific parameters including mean, dispersion, and zero-inflation parameters [13,24,32,33]. For DEA of genes (two cell groups’ simple comparison), the models in Equations (2) and (7) can be written as:

(9) $$log\mu_{ij}=\beta_{i0}+\beta_{i1}x_{ij}+O_{\mu }$$

where, $\beta_{i0}$: intercept term; $\beta_{i1}$: regression co-efficient for cell group; and, $O_{\mu }$**:** offset term. The model in Equation (9) can also be expanded to accommodate other cell-level co-variates including cell type, cell cycle, cell growth phase, etc., [13]. To test whether the *i*th gene is differentially expressed or not across the cell groups, GLM-based approaches test the following null hypothesis:

$$H_{0}:\;\beta_{i1}=0\;\mathrm{vs}.\;H_{1}:\;\beta_{i1}\neq 0$$

Importantly, all GLM-based approaches use the likelihood ratio test (LRT) statistic. Mathematically, the LRT statistic (for *i*th gene) is −2*logL* (*L*: likelihood function) (i.e., deviance divided by the scale parameter *ϕ* called scaled deviance) which can be expressed as:

(10) $$-2logL\left(\Omega_{i}|y_{ij}\right)=\frac{1}{\phi }D\left(y_{ij}|\Omega_{i}\right)=2l\left(y_{ij}|y_{ij}\right)-2l(\Omega_{i}|y_{ij})\sim \chi_{\left(M-p\right)}^{2}$$

where, $l(\Omega_{i}|y_{ij})$: log-likelihood function; and $l\left(y_{ij}|y_{ij}\right)$: log-likelihood function for the saturated model (i.e., maximum likelihood achievable when the fitted values are exactly equal to the observed data for exponential family distribution). The test statistic in Equation (10) follows a Chi-square distribution with certain degrees of freedom.

Additionally, modifications in the GLM have been performed by adding random components for different cell-level factors to build generalized linear mixed models. Recently, such models have been implemented in two-part mixed model (TPMM) and scMMST approaches.

**Limitations**: There are three major limitations of this class of approaches. Firstly, *strict model assumptions*: the GLM class of approaches requires several distributional assumptions about the expression counts, which may not be satisfied by the real single-cell data. For instance, GLM requires the counts to be generated by exponential family distributions; the link function must be invertible, continuous, and differentiable; and it linearly depends on cell co-variates. These strict assumptions restrict the utility of GLM-based DEA approaches for real data analysis. In most cases, the users simply apply these techniques without testing or violating these assumptions, which causes the results to be misleading.

For the second limitation, *multi-modality,* several previous studies [37,38,45,47,64,65,66,67] report multi-modal distributions of scRNA-seq gene expressions, which may be due to a gene’s expression deriving from multiple cell states or from a series of biological processes [65]. For instance, a cancer-suppressor gene is over-expressed (i.e., higher counts) in some cells and its expression is suppressed (by its regulator genes) causing low expression in other cells. This negative feedback causes oscillations in gene expression across the cells [64], leading to multiple modes in scRNA-seq data [67]. The NB-GLM approaches fail to handle the multi-modal distribution of scRNA-seq data, while the ZIM–GLM-based approaches are able to tackle the bio-modality (i.e., modes due to biological zeros and non-zero counts) of underlying data. In other words, GLM-based tools cannot handle the multi-modality (>2) of expression counts distribution, which is inherent to scRNA-seq studies.

For the third limitation, *computational complexity*, this class of tools is computationally intensive due to implementation of iterative techniques for parameters estimation. For instance, DECENT, SwarnSeq and ZingeR, etc., approaches usually take more than 10–12 h to analyze the scRNA-seq data (with a few thousand genes and hundreds of cells) [10]. In addition, the EM algorithm employed in these tools fails to converge in most of the genes, causing the computational process to be slow and cumbersome. Furthermore, complex statistical models are fitted for each gene individually in a large dataset, making the implemented tools’ runtime inefficient. Further features and limitations of this class of tools are listed in Table 2.

### 3.2. Generalized Additive Model-Based Approaches

**Figure 4:**
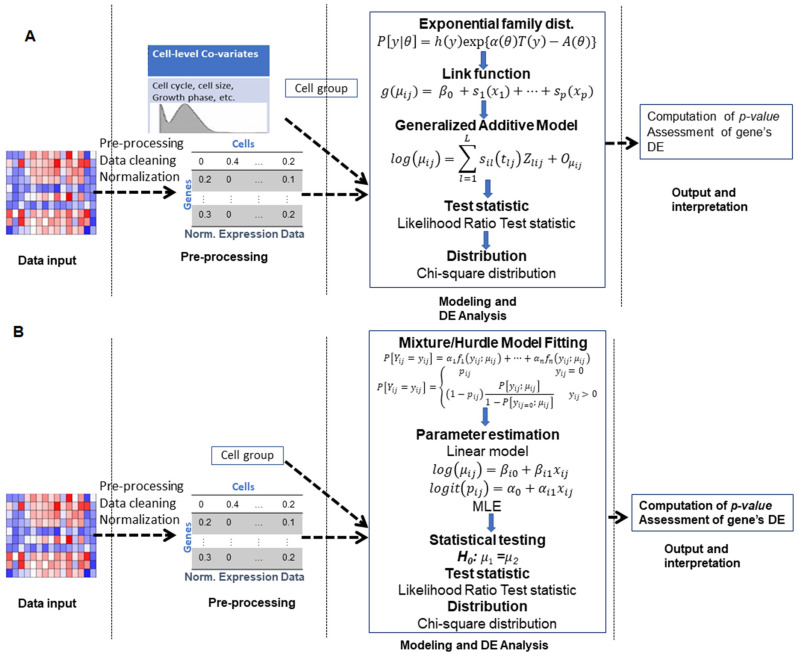
Operational outlines of DE analytic GAM, Hurdle and mixed model class of approaches in scRNA-seq studies. (**A**) Workflow of steps for GAM-based DEA approaches. (**B**) Workflow of steps for Hurdle and mixed-model-based approaches. In both classes, the framework can be divided into four major parts, namely: (i) input (data provided as input to tools); (ii) pre-processing of data, this step involves data cleaning, outlier removal, normalization, etc.; (iii) model fitting and computation of DEA test statistic, various distributional/model (e.g., GAM, Hurdle or mixture model) assumptions are made about the expression data, parameters of the models are estimated, DEA test statistic(s) for genes and their corresponding *p*-values are computed; and (iv) assessment and interpretation of DEA results.

Generalized additive models (GAM) are natural extensions of the GLM, where the link function is additive but each term/predictor non-linearly depends on the mean and zero-inflation parameters of the gene. GAMs are similar to GLMs but allow testing of variables in response to a numerically estimated trend in the predictors, alleviating the burden of specifying their distribution. While this necessitates some approximations in downstream testing, it has proven to be highly effective in many settings, particularly when one wishes to model the response variable as a function of both categorical (e.g., cellular group) and continuous (e.g., cell growth phase, cycle, etc.) predictors. The operational framework of this class of approaches is shown in Figure 4.

The GAM uses smooth (non-parametric (NP)) spline functions to capture the relationships between individual cell co-variates and the expression of gene, which can be linear or nonlinear. In other words, these smooth relationships can be simultaneously estimated, and then, the expected expression values predicted, by simply adding them. The GAM class of DEA approaches includes Monocle [37], Monocle2 [38], and tradeSeq [39], etc.

The impact of the predictive variables is captured through smooth functions, depending on the underlying patterns in the data, which can be nonlinear:

(11) $$g\left(\mu_{ij}\right)=\beta_{0}+s_{1}(x_{1})+s_{2}\left(x_{2}\right)+\;\ldots +s_{p}\left(x_{p}\right)\;$$

where, $x_{l}$: cell level co-variates/predictors; and $S_{il}\left(.\right)$: smooth function.

The GAM-based approaches use a *log* link function that depends on the pseudo-time of the cell, as shown in Equation (12).

(12) $$log\left(\mu_{ij}\right)=\sum_{l=1}^{L}s_{il}\left(t_{lj}\right)Z_{lij}+O_{\mu_{ij}}\;$$

where, $s_{il}()$: smooth function for *i*th gene at *l*th cell lineage, which are functions of pseudo-time $t_{lj}$, ∀lϵ{1, 2,…, L}; and, $Z_{lij}:$ binary variable for gene expression, i.e., $Z_{lij}=1;\;y_{lij}>\tau \;or\;0\;else$ (τ: hard threshold). The smoothing spline function in Equation (12) can be represented as a linear combination of *K* cubic basis functions ($b_{k}\left(.\right)$).

(13) $$s_{il}\left(t_{lj}\right)=\sum_{k=1}^{K}b_{k}\left(t\right)\beta_{ilk}$$

For testing *i*th gene at *l*th cell lineage, the null hypothesis, $H_{0}:\beta_{ilk}=0$ is tested for its possible rejection through the Wald test statistic (e.g., tradeSeq) or LRT (e.g., Monocle). Further, Monocle (also Monocle 2, 3) only considers one cell lineage (i.e., L=1) while tradeSeq considers multiple cell lineages (i.e., L≥2)). Additionally, the latter provides provision for the DEA of genes within and across cell lineages. The special features of the GAM-based approaches are listed in Table 2.

**Limitations**: (i) *Pseudo-time dependent*: Approaches including Monocle heavily depend on the accuracy of the pseudo-time-ordering of cells. In other words, in single-cell studies, expression of genes in each cell is a function of time, therefore, cells can be ordered by the time. Single-cell analytical tools use existing algorithms including Wanderlust [68] to order the single-cells along discrete paths. These paths do not represent real time but rather a pseudo-time variable (due to short life cycles of cells), which usually represents the intrinsic cellular process. Further, computational experiments indicate that differences in the temporal ordering of the single-cells from different approaches affect the results, and thus interpretations [69]. The use of pseudo-temporal ordering along with expression data has been useful in some studies, but it has also faced criticism. For instance, Moris et al. 2016 [70] questioned the underlying assumptions of smooth and continuous cell state transitions, which are required by pseudo-time-ordering algorithms. Moreover, such data may not be readily available for the users, thus making it difficult to apply in general cases; (ii) Similar to the GLM classes of approaches, this class is also unable to consider the multi-modal nature of single-cell data; and, (iii) The GAM class of approaches is computationally intensive, due to implementation of complex statistical models fitted individually for each gene.

### 3.3. Mixture Model-Based Approaches

The observed gene expressions in a scRNA-seq experiment are the noisy reflections of true expression levels due to various biological and technical sources. Hence, the mixture model (MM) framework (shown in Figure 4) assumes that the distributions of the observed expression counts are decomposed into multiple parts or a mixture of probability distributions, as shown in Equation (14):

(14) $$P\left[Y_{ij}=y_{ij}\right]=\alpha_{1}f_{1}\left(y_{ij};\Omega_{ij}\right)+\alpha_{2}f_{2}\left(y_{ij};\Omega_{ij}\right)+\ldots +\alpha_{n}f_{n}\left(y_{ij};\Omega_{ij}\right)$$

where, $f_{1}\left(.\right),\;f_{2}\left(.\right),\ldots ,\;f_{n}\left(.\right)$ are the probability distributions associated with various components of single-cell studies, e.g., dropout events, amplification, etc.; and $\alpha_{1},\;\alpha_{2}\ldots ,\alpha_{n}$ are the corresponding weights of the distribution functions such that $\alpha_{i}>0\;\mathrm{and}\;\sum_{i=1}^{n}\alpha_{i}=1$. In other words, the PMF of the expression counts is expressed in terms of the linear combination of the distribution functions of various components of single-cell studies (a convex combination).

The MM class includes popular approaches and tools such as SCDE [42], D3E [43], BPSC [12], BASiCS [25], DESCEND [28], and SC2P [44], etc. For instance, SCDE models the expression (in terms of reads per million) of *i*th gene in *j*th cell using a mixture of Poisson and NB distributions, and the PMF for the SCDE can be written as:

(15) $$P\left[Y_{ij}=y_{ij}\right]=\alpha f_{NB}\left(y_{ij};\mu_{ij},\theta_{ij}\right)+\left(1-\alpha \right)f_{PD}\left(y_{ij};\gamma_{ij}\right)$$

where, $f_{NB}$ and $f_{PD}$: PMF of NB and Poisson distributions, respectively; $\gamma_{ij}$: parameter of Poisson distribution; and α=log(e) (*e*: expected expression magnitude).

Further, approaches such as BPSC and D3E use the Beta-Poisson model to fit the expression counts of genes in scRNA-seq data to capture the cell burst size and burst frequency of the gene level expression data. However, the BPSC uses a linear model framework (to model $\mu_{ij}$ through log-link function) to perform DEA, while D3E employs three statistical tests, i.e., Cramer–von Mises test, KS test or the LRT for differential expression testing of genes. It is interesting to note that BPSC can be generalized to multi-cellular group comparison, while D3E is limited to only two group comparison. Moreover, the DESCEND, BASiCS, SC2P tools use the Poisson-Alpha, Poisson-Gamma, and Poisson-Lognormal MMs, respectively, to fit the observed scRNA-seq data.

Additionally, approaches including ZIAQ [45] and ZIQRank [47] use logistic regression and quantile regression to model the dropout events/zero-inflation and non-zero expression counts (shifted quantiles), respectively [47]. It is worthy to note that this class of approaches uses different setups to perform DEA of genes across the cell groups. For instance, ZIQRank and ZIAQ use the Cauchy and Fisher test, respectively, to compute the *p*-values for genes [45,47]. The major pros and cons of this class of approaches are listed in Table 2.

### 3.4. Hurdle Model-Based Approaches

In Hurdle model-based approaches, the expression counts of genes are modeled in two parts, namely, (i) zero counts ($\left[W_{ij}=0]\sim f_{1}\left(;\theta_{i}\right)\right)$ and (ii) non-zero counts ($Y_{ij}|W_{ij}=1\sim f_{2}\left(;\theta_{i}\right)$). In other words, the first part of the model fits the zero counts, while the second part models the probability of the non-zero expression values (through a truncated (at zero) probability distribution function). Mathematically, the PMF of the Hurdle model is shown in Equation (16):

(16) $$P\left[Y_{ij}=y_{ij}\right]=\{\begin{array}{c} p_{ij}\;\;y_{ij}=0\\ \left(1-p_{ij}\right)\frac{P\left[y_{ij};\mu_{ij}\right]}{1-P\left[y_{ij=0};\mu_{ij}\right]}\;\;y_{ij}>0 \end{array}$$

where, $p_{ij}$: probability of expression counts of *i*th gene in *j*th cell belongs to the zero component.

Statistically, the ZIM and Hurdle models differ based on their conceptualization of the zeros in scRNA-seq data and interpretation of model parameters. In other words, the ZIM model always assumes that zero counts are derived from a mixture of two distributions: (i) the first part produces zero counts which are often called “structural zeros” or “excessive zeros” (e.g., absence of mRNA of a gene) (modeled using Dirac’s delta function); and, (ii) the second part produces zero counts termed as “sampling zeros” (e.g., absence of expression counts due to inefficient amplification/limited sequencing depth) (modeled using the count data model).

The Hurdle model-based approaches model the gene parameters using linear models, as shown in Equations (17) and (18).

(17) $$log\left(\mu_{ij}\right)=\beta_{i0}+\beta_{i1}x_{ij}$$

(18) $$logit\left(p_{ij}\right)=\alpha_{0}+\alpha_{i1}x_{ij}$$

For the DEA of *i*th gene, the null hypothesis is tested for its possible rejection using the test statistic given in Equation (10). This class includes popular approaches such as MAST [40] and Random Hurdle [41]. Further, the MAST approach uses a logistic regression model to fit the indicator variable (for zeros), $W_{ij}$ and the Gaussian linear model for the continuous variable (non-zero expression values) $\left(Y_{ij}|W_{ij}=1\right)$, independently. The unique features of this class of approaches are listed in Table 2.

The major limitations of the Hurdle model-based approaches are: (i) The Hurdle model considers the sources of zeros in single-cell studies without making any distinctions. In other words, the Hurdle model can have restrictive assumptions that fail to distinguish between structural zeros and sampling zeros [8], which can be quite detrimental when the assumptions are violated. By design, the Hurdle model will always predict the same number of zeros as observed in the scRNA-seq data without telling their sources; (ii) Several of these approaches require a data transformation including the use of a pseudo-count and log-transformation, but this has recently been shown to introduce false variation in downstream analyses [71,72]; and, (iii) These approaches are computationally intensive especially for large single-cell datasets, as they fit models individually for each gene, such as GLM-based approaches. Further limitations are listed in Table 2.

### 3.5. Two-Class Comparison (Parametric) Approaches

The above four classes of approaches consider the inflated zero counts inherent to the scRNA-seq data through various provisions in the underlying statistical models. However, they use complex linear models to perform DEA of genes, which require more computational time to individually fit the models for each gene. Therefore, another class of parametric approaches is reported in the literature, which is straight forward. In other words, this class of approaches is quite simple to execute, as they compare the mean expressions/estimated parameters of genes across two cell groups/populations. Further, their modes of execution require two simple steps: (i) estimation of gene-level parameters including mean, dispersion, etc., using a parametric model; and, (ii) comparison of the estimated mean parameters of genes between the two cell groups through a parametric statistical test. The operational procedure for this class of approaches is shown in Figure 3.

This class of approaches includes, scDD [50], DEsingle [51], NYMP [52], IDEAS [53], and, *t*-test [10], etc. For instance, DEsingle assumes the UMI counts must follow ZINB distribution [51], as given in Equation (4), but does not use GLM framework to model the mean parameter. Further, DEsingle uses the EM algorithm to estimate the parameters of the ZINB model across the two cell types, while scDD utilizes a conjugate Dirichlet normal distribution to fit the expression data, thus handles the hidden cellular heterogeneity. The underlying statistical models and unique features of the other approaches are listed in Table 1.

The underlying null hypothesis for this class of approaches can be expressed as:

(19) $$H_{0}:\mu_{i1}=\mu_{i2}\;\mathrm{vs}.\;H_{1}:\mu_{i1}\neq \mu_{i2}$$

where, $\mu_{il}$ is the mean expression of *i*th (*i* = 1, 2, …, *N*) gene in *l*th (*l* = 1, 2) cell populations/groups.

This class of approaches statistically tests the estimated value of the mean parameter of genes across the two cell populations using various test statistic(s) [10,16,51]. For instance, DEsingle uses the LRT (following a Chi-square distribution), while the scDD uses the Bayesian posterior probability. The test statistic(s) for other approaches is given in Table 1. Though this class of approaches is simple and quick in terms of execution, it suffers from serious limitations.

**Limitations**: (i) *Only two groups*: This class of approaches cannot be generalized to accommodate multiple cellular groups, though it is clear that scRNA-seq data are characterized by the presence of multiple cell types/groups, which these methods are unable to consider. This is due to the fact that other classes of methods including GLM, GAM, Hurdle, and MM, consider the GLM to model the mean parameter of genes, which can accommodate the multi-group comparison; (ii) *Cell-level auxiliary data*: The incorporation of cell-level confounding covariates including cell type, cell cycle, cell growth phase, etc., in the DEA improves the statistical power to detect true differentially expressed genes in single-cell studies. Therefore, this class of approaches cannot accommodate such auxiliary data in the analysis, and thus has poor performance compared with other class of approaches [10,16]; and, (iii) Many aforementioned approaches consider the inflated zero counts through parametric models (e.g., ZINB) which might not be sufficient to capture the heterogeneity in the scRNA-seq data. Further limitations and unique features of this class of approaches are listed in Table 2.

### 3.6. Non-Parametric Approaches

The approaches described in the previous five classes assume that the expression counts follow a well-defined parametric distribution. These approaches are typically slower due to the implementation of complex statistical models and iterative algorithms of parameter estimation. Thus, parametric models may not be ideally suited to data from many hundreds or thousands of single-cells [47,56,60]. Furthermore, parametric tests including LRT, Wald test, etc., have been used to compute the statistical significance of differentially expressed genes across the cell population. However, in statistics, NP methods have statistical power at par or greater than parametric methods, if the data violate the underlying assumptions of the parametric methods and a large number of samples/cells exist [73]. Under these circumstances, NP approaches can be better alternatives to their parametric counterparts, and are ideally suited to large single-cell datasets [74]. Further, the NP class of approaches can capture the multi-modal nature of the single-cell data. The major pros and cons of this class of approaches are described in Table 2.

The NP class of approaches includes NODES [56], Wilcoxon signed rank test (Wilcox) [10,16], ROTS [75], EMDomics [57], ROSeq [60], and SINCERA [55], etc. This class of approaches estimates the parameters that can quantify the distribution of expression profiles and makes comparisons between two cell groups. The null hypothesis of this class of approaches can be expressed as:

$$H_{0}:F_{i}=G_{i}\;vs.\;H_{1}:F_{i}\neq G_{i}$$

where, $F_{i}\;\mathrm{and}\;G_{i}$ are the distributions of *i*th gene in the first and second cell groups, respectively. The above null hypothesis indicates that the two cell populations have the same distributions or derive from a same cell population. For instance, NODES and Wilcox usually test significant differences of genes’ mean expressions across the two cell groups [10,16]. The former requires pseudo-counted quantile normalized gene expression values for DEA, while the latter can be used for counts or normalized data. Further, these approaches use test statistic(s) based on ranks (e.g., Wilcox, ROSeq), quantiles or percentiles (e.g., NODES), or distance measures (e.g., EMDomics), etc., for DEA of genes. For instance, Wilcox compares the ranks of the expression values that derive from the two cell groups. This rank-based test mostly ignores the magnitude of the expression deviations of genes between the two cell groups. Moreover, this class of approaches mostly uses permutation or bootstrap procedure to compute the *p*-values for genes (e.g., NODES [56], ROTS [75]). These approaches are relatively simple to understand and easy to execute for large scRNA-seq datasets with relatively lesser runtime required, compared with the other five classes.

**Limitations**: (i) *Lesser statistical power*: If all of the assumptions of the parametric approaches are apparently met by the single-cell data, and the DEA hypothesis can be tested with a parametric approach, then NP approaches may not be suitable. The degree of unsuitableness can be expressed in terms of lesser statistical power. Previous studies indicate that ZIM (Supplementary Documents S3–S5) fits well to the single-cell data [13,34]. Subsequently, DEA approaches based on ZIM usually have better performance over NP approaches [10,16]; (ii) NP approaches are not systematic, whereas parametric approaches have been systematized, and different tests are simply variations on a central theme; (iii) Another objection to NP approaches is related with convenience. Tables necessary to implement NP tests are scattered widely and appear in different formats; and, (iv) The results may or may not provide an accurate answer because they are distribution free. Further limitations and special features of this class of approaches are listed in Table 2.

## 4. Outstanding Challenges

The challenges in DEA of scRNA-seq data can be divided into two broad categories: (i) biological challenges, and (ii) methodological challenges.

### 4.1. Biological Challenges

We believe that development of the DEA approaches will require improvement of the existing annotations of genes on real single-cell data applications. Therefore, it is necessary to create accurate, high resolution knowledge bases with detailed annotations of genes. These knowledge bases will help investigators assess their DEA approach’s performance based on the biological ground truth, and will also help in understanding the biological process from a systems biology point of view.

#### 4.1.1. Proper Biological Benchmarking

Simulation techniques are usually used to validate the performance of the DEA approaches in scRNA-seq studies [11,14,16,17,18,24,51]. In simulation studies, the ground truth (reference genes) is (artificial) known and biological data are mimicked through statistical models. Then, these artificial single-cell data are used to assess the performance of the statistical approaches, given the artificial reference genes. In other words, DEA approach’s performance is assessed through comparing the obtained differentially expressed genes with the given reference genes using sensitivity–specificity-based performance metrics [10,11,12,13,14,15,16,17,18,24,33,34]. However, Glazko et al. (2009) showed that statistical method’s performance on simulated and real biological data are significantly different [76], which raises several questions about the performance assessment of methods using simulation as a benchmark. The attributable reason may be that the biology is more complicated than artificial scenarios and is influenced by factors such as the absence of an exclusive division into classes, presence of outliers, biological or technical hidden factors, environmental influence(s), and random errors, etc. This aspect of performance evaluation is highly questionable among stakeholders, and needs further exploration.

To tackle this issue, researchers started using reference genes from microarrays [13,17,42] and bulk RNA-seq [10,13,24] (for the same bulk cell lines) to validate the performance of the DEA approaches on real single-cell data. However, this technique of performance validation has faced many criticisms from researchers, as scRNA-seq is the latest technology and obtaining reference genes from the old techniques incurs a technical lag. Therefore, to assess the performance of DEA approaches, proper biological benchmarking platform is required in single-cell studies.

#### 4.1.2. Annotation

Biology-based techniques have been utilized to assess the performance of DEA methods in microarrays data analysis [21,22]. In other words, the genes from microarrays are validated using bio-knowledge bases (KEGG, STRING, GO terms, pathways, QTLs, etc.) in order to find biological processes and pathways that are relevant to the underlying condition [77]. For instance, the QTLs, GO, etc., tools were previously used to validate the performance of DEA methods in microarrays under a biological framework [77,78]. Further, these bio-knowledge bases were well established for microarrays and RNA-seq studies [77,78]. The scRNA-seq technique has shifted the paradigm of gene expression dynamics to the single-cell resolution-level. Therefore, the current annotation databases need to be updated with respect to these high-resolution techniques. It is essential that they also begin specifying gene and cell level annotation information. Such information will provide a better platform for assessing scRNA-seq DEA approaches from biological perspectives.

In addition to annotation, other information including literature support and expert interpretation can be considered while assessing DEA approaches. Further, statisticians and bioinformaticians must work closely with experimental biologists to validate their in-silico findings in wet-lab conditions.

### 4.2. Methodological Challenges

In addition to the above biological challenges, we also highlight the methodological challenges involved in DEA of scRNA-seq data.

#### 4.2.1. Gold Standard scRNA-seq Data

The huge availability of statistical approaches for DEA of scRNA-seq data has prompted the search for methods which produce biologically accurate results. To address this, computational biologists have turned to simulations to mimic the biological ground truth through which DEA approaches could be benchmarked. Further, simulations require the specification of a model through which scRNA-seq data are generated. Differences in model parameters specifications have led researchers to generate irreproducible results [79]. These lacunae motivate for requirement a sound epistemological framework for DEA of scRNA-seq data [79,80]. To address this, Squair et al. (2021) suggested the quantification of performance of the DEA methods across multiple datasets in which the experimental ground truth was known, and also identified the principles/factors responsible for their performance differences [79]. This framework of gold standard data (i.e., data with known biological ground-truth) may provide a suitable platform for assessing the performance of the DEA approaches from a biological perspective.

#### 4.2.2. Excess Heterogeneity

The scRNA-seq data tend to have abundance of zero counts, complicated underlying distributions, and huge heterogeneity. Subsequently, the heterogeneity between and within cell populations poses greater challenges to the DEA of scRNA-seq data [53]. Further, this cellular heterogeneity in data will increase multifold if the single-cell data are collected over individuals/patients. Previously, researchers have usually considered bulk RNA-seq methods for DEA of scRNA-seq data, which may not be sufficient to handle the huge heterogeneity in the data. The implemented models in the existing single-cell DEA approaches (Supplementary Document S1) could best solve cellular heterogeneity. However, this may not be sufficient to tackle the heterogeneity in the data for single-cell studies over individuals/patients. Therefore, novel statistical approaches and tools are required for DEA of highly heterogeneous scRNA-seq data.

#### 4.2.3. Dropouts or Excess Zeros of Single-Cell Data

Existing approaches of bulk RNA-seq DEA have been optimized for bulk tissue samples, and either perform poorly on single-cell data or do not accommodate the special features brought out by the revolutionary single-cell technology [81]. The zeros in scRNA-seq data are mainly due to biological and non-biological sources, a well-known challenge in scRNA-seq data analysis, and how to best tackle it remains a controversial topic. It is very difficult to distinguish between biological and non-biological zeros in scRNA-seq data without pre-defined knowledge or spike-in control [8]. Therefore, researchers started using ZIM or Hurdle models to tackle the issue of zero-inflation or excess zeros [8,13]. For instance, the Hurdle model failed to consider the sources of zeros, and assumed them to be from a single source. A significant portion of these zeros is due to dropout events, which need to be addressed in the modelling process for better DEA. Another strategy of handling the dropout/false zeros (attributed to inefficient sample preparation and sequencing protocol) is through suitable data imputation tools (e.g., scImpute and DrImpute, etc.) [82]. Furthermore, lower transcriptional captures in single-cells also contribute to dropout events in the data. For instance, efficient protocols of single-cell sequencing can capture 1–10% of the transcriptomes present in the cell [13,24,83]. Hence, different capture rate models including Binomial and Hypergeometric, etc., [13,24] can be used to adjust the cellular capture rate while modelling the observed UMI counts. These efforts will help mitigate the issues associated with singe-cell data that limit the utilization of existing DEA approaches.

#### 4.2.4. Pre-Processing of scRNA-seq Data

The DEA seems to be a single-step process, but actually, unavoidably, is a multi-step process, and its success highly depends on the pre-processing of scRNA-seq data. For instance, scRNA-seq data usually have poor quality or outlier cells, which may bias the analytical findings if included in the analysis. Therefore, researchers remove the cells whose library size lies below a certain threshold [24], which is an empirical approach and does not consider the statistical distributions of the cell library sizes. Hence, bioinformatics tool developers must consider the pre-processing steps applied to input data, and the DEA may be performed on the processed data. For example, the popular Seurat package uses many data pre-processing steps before DEA of genes [48]. These pre-processing steps include filtering low-quality genes and cells, data normalization, pre-feature selection, dimensionality reduction, and cell clustering [49]. Hence, if DEA tool developers are not aware of these pre-processing steps, their bioinformatics tools may not identify true differentially expressed genes. In other words, accuracy and reproducibility of the DEA tool will depend on pre-processing of scRNA-seq data. Therefore, tool developers must consider data pre-processing as an integral part of the DEA for real data applications.

#### 4.2.5. Lack of Biological Relevant Criteria

The performances of the scRNA-seq DEA approaches are usually assessed on simulated data through sensitivity–specificity-based criteria. Though these criteria are statistically strong, they fail to state the biological relevance of the stated approaches. To address this, biologically relevant criteria (based on GO, QTL, and pathways, etc.) under a sound statistical framework have been developed for microarrays studies [21,77,78]. However, such comparative indices are missing in single-cell data analytics. In other words, such an assessment will answer the question of whether the differences between DEA approaches can impact the functional interpretation of transcriptomics experiments. Hence, Squair et al. (2021) used the GO term enrichment analyses in bulk vs. scRNA-seq DEA to assess the biological relevance of approaches [79]. However, strong statistical criteria are required for this purpose, based on biologically relevant information including GO, QTL or pathways information for single-cell datasets.

#### 4.2.6. Statistical Methods for DEA across Individuals

The standard practice in DEA of scRNA-seq studies is to collect many cells from one or a few individuals, and finding differentially expressed genes through the comparison of gene expression between two cell groups/clusters. Several methods have been developed for this purpose [10,11,16,24,51]. Currently, the scRNA-seq technique is slowly becoming a standard practice, and many investigators have started generating scRNA-seq data from multiple individuals. Thus, the DEA of genes across groups of individuals (i.e., comparison between case and controls) has opened new avenues, which requires novel and innovative statistical approaches and tools. The existing DEA approaches are inappropriate for individual level differential expression testing, as the sampling units for these approaches are cells, not individuals. For this purpose, IDEAS [53] is the only recently developed technique which performs DEA of genes across individuals, by capturing their cell type-specific gene expressions. However, computational biologists and bioinformaticians may focus on developing novel approaches and tools using multi-level hierarchical linear models.

#### 4.2.7. False Discoveries in DEA

If a method fails to account for cell–cell variations in DEA, then it could produce false discoveries in the presence of a real biological perturbation. The false discoveries may also arise in the absence of any biological difference. For instance, recent computational studies confirmed that single-cell methods produced a systematic excess of false positives compared with the bulk of RNA-seq DEA methods [79]. In addition, they found that the genes falsely identified as differentially expressed corresponded to those with the highest variability between replicates [79]. This exposes a fundamental pitfall for DEA in single-cell transcriptomics. In other words, the single-cell studies, especially in human, would exhibit greater variability between biological replicates, and consequently would be more vulnerable to false discoveries in DEA. These false discoveries are poised to mislead investigators. Therefore, novel and innovative statistical approaches and tools are of paramount importance to address this issue in DEA of scRNA-seq data.

#### 4.2.8. Improved Methods for Dispersion Estimation

In most of the DEA tools including DECENT, DEsingle, and SwarnSeq, etc., the MLE method has mostly been used to estimate the dispersion parameter through iterative algorithms (e.g., EM and ECM) [13,24,34,51]. The dispersion parameter represents the cellular variability, thus obtaining its good estimate is crucial to finding the true differentially expressed genes. For this purpose, Empirical Bayes (EB) shrinkage estimation using weighted conditional log-likelihood method was used in bulk RNA-seq DEA methods [84]. This type of estimate shrinks the dispersion estimates toward a common prior, instead of shrinking them completely to the common dispersion. Therefore, any forms of the EB method in the estimation of dispersion parameter may be incorporated in approaches including DECENT, SwarnSeq, ZINB–WaVE, and ZingeR, to have better performance. Given that scRNA-seq data are very sparse, it may be expected that there is potential benefit in using the EB to improve the existing approaches performance. Such an attempt will stabilize estimates of the gene-specific dispersion parameter. For instance, MAST [40] used the EB to shrink the gene-specific variance parameter. Therefore, it is imperative to implement the EB or equivalent methods within scRNA-seq DEA approaches to stabilize the dispersion, for better analysis.

#### 4.2.9. Random/Mixed Effect Models

The statistical models implemented in scRNA-seq DEA tools assume various factors (e.g., cell group and cell-level auxiliary variables) affecting the gene parameters, including that mean and zero-inflation have fixed effects [13,24]. For instance, DECENT and SwarnSeq assume the cellular groups, cell types and cell-level auxiliaries, etc., have fixed effects on gene mean and zero-inflation. The trend is the same for all of the developed approaches. Sometimes, these assumptions are necessary for methodological derivations, but are highly unrealistic in biology, as some factors may have random effects. This is due to the fact that cell biology is a highly dynamic system, and factors affecting genes’ expression have random/mixed effects. Therefore, researchers may think of implementing random or mixed effect models in DEA approaches, for better results.

#### 4.2.10. Optimal Combination of Algorithms

Previous studies have shown that no statistical approach performed better for all the single-cell datasets [10,11,14,15,16,17,18]. It has even been found that some bulk RNA-seq DEA methods performed at par, or even better, compared with their single-cell counterparts [10,16]. In other words, statistical tools have their limitations and distributional assumptions about the data, which make them sensitive to real data applications. Thus, the differentially expressed genes identified by them are quite different from each other [10], making them data dependent, which leads to unstable and sometimes inaccurate results [61]. To tackle this issue, a more reliable strategy is to apply all methods at hand, and form a community prediction for better analytical findings. Alternatively, combining an assortment of different DEA approaches can be a better choice for finding true genes from single-cell data. For instance, Li et al. (2022) developed an ensemble learning-based computational framework to produce more stable and accurate results through combining results from 12 individual approaches [61]. This finding has opened the quest to obtain the optimal combination of state-of-the-art individual approaches, for better results. This aspect of obtaining better results through combining algorithm(s) is at infant stage, and more computational studies are needed in single-cell studies. We have listed the strengths and weaknesses of each class of methods in Table 2. A natural extension may be that a suitable combination of approaches can be a good strategy for the finding of true differentially expressed genes, as the methods may mask each other’s weaknesses.

#### 4.2.11. Integration of Multi-Omics Data

Single-cell multi-omics profiling technologies are rapidly evolving, bringing newer techniques to improve our understanding of the unique function of the basic atom of life. Recently, progress has been made in single-cell analytics to more accurately detect cell types, performing downstream analyses, correcting technical sources of error, and delineating cell lineages and cell-state transitions, etc., [17,18,69,85,86,87,88,89,90,91]. For instance, other high-throughput genomic studies, including genome-wide association study, have emerged as a powerful approach to identify risk variants; hence, such data can be integrated with scRNA-seq data for better identification of true marker genes.

In this direction, a computational framework has been developed to integrate association data with scRNA-seq data for the identification of novel cell types and marker genes in COVID-19 infected patients [92]. Such integrated data require advanced tools for DEA. Additionally, other single-cell cross platform datasets are available due to the advancement of genomic technologies, which can be integrated with scRNA-seq data for identifying true biological differentially expressed genes. For instance, fluorescence in situ hybridization (FISH) methods [93] provide data on the spatial distribution of single-cells, which can be used as priors in the modelling of gene expressions, preferably through a Bayesian approach.

Another advantage of integrating FISH data with sequencing data is that the former is extremely accurate and free from dropout events [93], which will compensate the high dropout events in the scRNA-seq. Further, in the absence of, or minimal, dropout events, it will be possible to accurately model the observed expression counts of genes. It is also possible to integrate phenotypic data of cellular activation with scRNA-seq data for effective modelling of gene expression. Broadly, integration of single-cell sequencing (e.g., scRNA-seq) approaches with high throughput single-molecule imaging (e.g., FISH) or GWAS has a better chance to identify true DE genes at single-cell level, which requires innovative statistical approaches.

#### 4.2.12. Slow Computational Processing

For DEA, one must consider the computational processing speed for large-scale scRNA-seq datasets, as one may not wait for several hours to obtain the results [10,13,24,51]. For instance, the Drop-seq-based system generates expression data of thousand(s) of genes over a large number of cells (e.g., from 10,000 to million cells), and the existing approaches fit complex statistical models individually to each gene, which costs a lot of computational time. Further, these approaches and tools employ complex iterative algorithms (e.g., EM, ECM) to estimate the gene specific parameters (most cases do not converge). Through computational experiments, it was found that methods including ZingeR, DECENT, DEsingle, and SwarnSeq, etc., require several hours for even a small dataset [10]. This situation will be more worrisome for real large experimental single-cell datasets. Additionally, researchers use the artificial setup with a small number of genes over hundreds of cells to assess the performance of their methods, which is far from the experimental reality. Thus, computationally efficient tools are required for the DEA of scRNA-seq data. Sometimes, to speed up the analysis, researchers consider pre-selected genes or dimensionality reduction technique, which restricts the analytical inference to prior knowledge or ignoring other important genes. Hence, future DEA approaches and tools must consider the scalability issue in single-cell studies.

## 5. Conclusions

DEA has become the primary downstream analysis of scRNA-seq data for extracting valuable biological insights into high-throughput gene expression measurements. This analysis also provides input to other secondary bioinformatics analyses including gene set analysis, gene network analysis, and pathways analysis, etc. To date, several statistical approaches and tools have been developed in the literature based on various statistical principles. This paper discusses the critical reviews of state-of-the-art methods available for DEA of scRNA-seq data, and distinctly classifies them into six major classes based on the underlying statistical models. Although the first three classes of methods, namely, GLM, GAM, MM, and Hurdle model approaches are extremely popular due to their ability to accommodate cell-level auxiliaries, they are computationally complex and runtime intensive.

Despite these developments, certain challenges exist in the DEA of scRNA-seq data, which need to be addressed in the future to develop improved classes of methods. We grouped the existing challenges of DEA into biological and methodological challenges. Under the biological challenges, a lack of proper biological benchmarking and incomplete annotations of genes in single-cell studies restrict the ability to assess the performance of DEA approaches for speaking the biological ground truth. We also reported several methodological challenges in DEA. The bioinformatics community must address these challenges to develop novel and innovative classes of DEA approaches and tools. These new approaches will utilize the features of the relatively new high-throughput single-cell technologies in order to better understand large biological systems.

## Figures and Tables

**Table 1:** Comparative overview of the DEA approaches for scRNA-seq data analysis.

SN.	Methods	Year	Model	Input	DE Test Stat.	Runtime	Platform	Ref.
1	NBID	2018	NB (GLM)	Counts	LRT	Medium	R code	[30]
2	ZINB–WaVE	2018	ZINB (GLM)	Counts	LRT	High	Bioconductor, GitHub	[31]
3	zingeR	2018	ZINB (GLM)	Counts	LRT	High	GitHub	[32,33]
4	DECENT	2019	ZINB (GLM)	Counts	LRT	High	GitHub	[24]
5	SwarnSeq	2021	ZINB (GLM)	Counts	LRT	High	GitHub	[13]
6	Tweedieverse	2021	ZITweedie (GLM)	Counts	Wald	High	GitHub	[34]
7	scMMST	2021	GLMM	Counts	Norm. score	High	NA	[35]
8	TPMM	2022	GLMM	Norm.	Wald/LRT	High	GitHub	[36]
9	Monocle2	2017	GAM	Norm.	LRT	Medium	Bioconductor	[37,38]
10	tradeSeq	2020	GAM	Counts	Wald	Medium	GitHub	[39]
11	MAST	2015	Hurdle	Norm.	LRT/Wald	Medium	Bioconductor	[40]
12	Random-Hurdle	2019	Hurdle	Counts	Chi-square test statistic	High	NA	[41]
13	SCDE	2014	Poisson-NB (MM)	Counts	Bayesian stat.	High	Bioconductor	[42]
14	BASiCS	2015	Poisson-Gamma (MM)	Norm.	Posterior prob.	High	Bioconductor	[25]
15	D3E	2016	Poisson-Beta (MM)	Counts	CM/KS test	High	GitHub	[43]
16	BPSC	2016	Beta-Poisson (MM)	Counts	LRT	Medium	GitHub	[12]
17	TASC	2017	Logistic, Poisson Models (MM)	UMI	LRT	High	GitHub	[26]
18	DESCEND	2018	Poisson-Alpha (MM)	Counts	Normalized Gini Score	High	GitHub	[28]
19	SC2P	2018	ZIP, Poisson-Lognormal (MM)	Counts	Posterior prob.	High	GitHub	[44]
20	ZIAQ	2020	Logistic and quantile Regression (MM)	Norm.	Fisher’s test	Medium	GitHub	[45]
21	SimCD	2021	Gamma-NB (MM)	Counts	Bayesian	High	GitHub	[46]
22	ZIQRank	2022	Zero-inflated model, quantile regression (MM)	Cont.	Rank-score test	High	NA	[47]
23	Seurat	2015	NB (TCP)	Counts	LRT	Low	CRAN	[48,49]
24	scDD	2016	Multi-modal Bayesian (TCP)	Norm.	Bayesian stat.	High	Bioconductor	[50]
25	DEsingle	2018	ZINB (TCP)	Counts	LRT	High	Bioconductor, GitHub	[51]
26	NYMP	2019	Logistic regression (TCP)	Cont.		Medium	GitHub	[52]
27	*t*-test		logCPM (TCP)	Norm.	T stat	Low	CRAN	[10]
28	IDEAS	2022	NB/ZINB/Kernel Density estimation/ Cumulative distribution function (TCP)	Counts/Cont.	Jensen–Shannon Divergence/ Wasserstein distance	High	GitHub	[53]
29	SAMstrt	2013	NP	Counts		Medium	GitHub	[54]
30	Wilcox		NP	Counts/Norm.	Sum ranks	Low	CRAN	[10]
31	SINCERA	2015	NP	Norm.	Welch (LS)/ Wilcox (SS)	High	GitHub	[55]
32	NODES	2016	NP	Norm.	Wilcox	Medium	Dropbox	[56]
33	EMDomics	2016	NP	Norm.	Euclidean distance	High	Bioconductor	[57]
34	sigEMD	2018	NP	Norm.	Distance measure	High	GitHub	[58]
35	DTWscore	2017	NP	FPKM	Distance	Medium	GitHub	[59]
36	ROSeq	2021	NP	Counts/Norm.	Wald	High	Bioconductor, GitHub	[60]
37	scDEA ^1^	2021	12 Models (Hybrid)	Counts	Lancaster’s test (Chi)	High	GitHub	[61]

CM: Cramér–von Mises test; Counts: read/UMI counts; Cont.: continuous values, e.g., FPKM, log(CPM), RPKM; NA: source codes are not freely available; Norm.: normalized; GLM: generalized linear model; NB: negative binomial; GLMM: generalized linear mixed model; NP: non-parametric; GAM: generalized additive model; MM: mixture model; TCP: two-class parametric. ^1^: Integrated approach.

**Table 2:** Classes of statistical approaches and tools extensively used in DEA of scRNA-seq data.

SN.	Class	Features	Limitations	Tools
1	GLM	Gene expression can have any form of exponential distribution type. Suitable for bi-modality of data. Able to deal with categorical predictors, e.g., cell type, cell cycle, etc. Easy to interpret and allows a clear understanding of how each of the predictors are influencing the gene parameters. Can be generalized to multi-cell group comparisons. Less susceptible to model over-fitting.	Strict exponential family distributional assumptions about the data. Needs relatively large datasets (with more predictor and large number of cells). Sensitive to outliers. Sensitive to dropout events. Not suitable for low expressed genes. Cannot handle multi-modality of the data. ZIM–GLM approaches are not able to handle zero-deflation at any level of a factor and will result in parameter estimates of infinity for the logistic component. Higher computational cost especially for large datasets.	NBID, ZingeR ZINB–WaVE, DECENT, SwarnSeq, scMMST, TPMM, Tweedieverse
2	GAM	Predictor functions are automatically derived during model estimation. Marginal impact of a single variable does not depend on the values of the other variables in the model. Flexibility in choosing the type of functions, which will help in finding patterns missed in a parametric model. Allows controlling smoothness of the predictor functions to prevent model over-fitting. By controlling the wiggliness of the predictor functions, we can directly tackle the bias/variance tradeoff. Highly effective in many settings, particularly when one wishes to model the response variable as a function of both categorical (e.g., cell groups) and continuous predictors (e.g., cell-level auxiliary variables). Considers both linear and non-linear functions of cell-level predictors to model gene parameters. Each lineage is represented by a separate cubic smoothing spline, and its flexibility allows adjustment for other covariates or confounders as fixed effects in the model.	Approaches such as Monocle can only handle a single lineage of cells. Lack of interpretability, to infer differences in expression between lineages of cells. Assumes the dropout events to be linear; however, the effect of dropout events is likely to be non-linear, especially for genes with low to moderate expression. Computationally complex.	Monocle, Monocle2, Monocle3, tradeSeq
3	Hurdle Model	Considers the excess zeros while model building. Can handle zero-inflation as well as zero-deflation present in data. Models the bimodality of gene expression distribution.	Does not differentiate the generating process for excessive zeros versus sampling zeros. Fails to consider the multi-modality of gene expression distribution. Requires higher runtime.	MAST, Random Hurdle
4	Mixture-Model	Considers bi-modal or multi-modal nature of single-cell data. Can differentiate between major sources of variation in single-cell data.	Certain approaches including BPSC, SC2P cannot consider the zero-inflation in single-cell data. Mostly uses linear models for DEA, which is cumbersome. Higher runtime and computationally intensive.	SCDE, D3E, BPSC, BASiCS, DESCEND, SC2P, ZIAQ, ZIQRank, SimCD
5	Non-parametric (two-class)	Distribution-free approaches. Considers the multi-modality of the data. Computationally not cumbersome (less runtime). Estimates the parameters without fitting any distribution for genes. Performs DEA with distance-like metrics across two cell types. Performs well when there are lesser proportions of zeros in the data.	Mostly focuses on two cellular groups’ comparison. Computationally complex for multi-groups. Performance severely affected due to high dropouts (some methods exclude dropouts). Cannot separate between true/biological and false/dropout zeros. Sensitive to sparsity. Methods such as D3E, scDD fail to consider UMI count nature of the data. Cannot separate confounding factors from each other.	Wilcox, NODES, ROTS, EMDomics, ROSeq, SINCERA, sigEMD, DTWscore, SAMstrt
6	Parametric (two-class)	Easy to understand and execute. Lesser runtime. Particularly suitable for larger datasets.	Makes strict distributional assumption about the data. Cannot generalize to multi-group comparisons. Ignores the multi-modal distributions of the scRNA-seq data. Sensitive to sparsity or dropout events. Cannot differentiate between the major sources of variability in the data.	scDD, DEsingle, *t*-test, NYMP, IDEAS

## Data Availability

Not applicable.

## Supplementary Materials

The following supporting information can be downloaded at: https://www.mdpi.com/article/10.3390/e24070995/s1, Supplementary Document S1: scRNA-seq DE Analysis Approaches; Supplementary Document S2: Count data models; Supplementary Document S3: Testing for zero inflation parameters for genes in scRNA-seq data; Supplementary Document S4: Statistical testing for overdispersion parameters in scRNA-seq data; Supplementary Document S5: Application of Count Data Models to Zero-Inflated and Overdispersed Real Datasets. References [94,95,96,97,98,99,100,101,102,103,104,105] are cited in the supplementary materials.
