# Supplementary material for: Differential Expression Analysis of Single-Cell RNA-Seq Data: Current Statistical Approaches and Outstanding Challenges
Source: Entropy (Basel). 2022 Jul 18;24(7):995. doi: 10.3390/e24070995 (PMC9315519; doi:10.3390/e24070995)
Supplement: Supplementary file 1 [file entropy-24-00995-s001.zip › entropy-1757058-supplementary.pdf]

Supplementary Materials

of

Differential Expression Analysis of single-cell RNA-Seq Data: Current Statistical Approaches and Outstanding Challenges

Samarendra Das[1-3*], Shesh N. Rai[4-9,*]

[1]ICAR-Directorate of Foot and Mouth Disease, Arugul, Bhubaneswar 752050, Odisha, India

[2]International Center for Foot and Mouth Disease, Arugul, Bhubaneswar 752050, Odisha, India

[3]ICAR-Indian Agricultural Statistics Research Institute, PUSA, New Delhi 110012, India

[4]School of Interdisciplinary and Graduate Studies, University of Louisville, Louisville, KY 40292, USA

[5]Biostatistics and Bioinformatics Facility, Brown Cancer Center, University of Louisville, Louisville, KY 40202, USA

[6]Biostatisitcs and Informatics Facility, Center for Integrative Environmental Health Sciences, University of Louisville, Louisville, KY 40202, USA

[7]Data Analysis and Sample Management Facility, the University of Louisville Super Fund Center, University of Louisville, Louisville, KY 40202, USA

[8]Hepatobiology and Toxicology Center, University of Louisville, Louisville, KY 40202, USA

[9]Christina Lee Brown Envirome Institute, University of Louisville, Louisville, KY 40202, USA

Authors' email addresses:

SD: samarendra.das@icar.gov.in, samarendra.das@louisville.edu

SNR: shesh.rai@louisville.edu

***To whom correspondence should be addressed**- email: shesh.rai@louisville.edu and samarendra.das@icar.gov.in.

1. <u>Document S1</u>. scRNA-seq DE Analysis Approaches

Notation: Y_{ij}: random variable (*rv*) represents observed read (UMI) counts of ith (i = 1, 2, …, N) gene in j^{th} (j = 1, 2, …, M) cell; N: total number of genes; M: total number of cells; μ_{ij}: mean of ith gene in j^{th} cell for NB distribution (count part of the model); θ_{ij} $(= \varphi_{ij}^{-1})$ and φ_{ij}: size and dispersion parameters respectively of ith gene in j^{th} cell for NB distribution; π_{ij}: mixture probability (zero inflation probability) of ith gene in j^{th} cell; s_j: size factor of jth cell; Z_{ij}: *rv* represents the true (unknown) concentration of reads for i^{th} gene of jth cell; $\boldsymbol{X}$: design matrix for cell group information, the jth row of $\boldsymbol{X}$, $X_j = [X_{j1}, X_{j2}, \dots, X_{jN}]$; W_{ij}: indicator *rv* representing the rate of expression for i^{th} gene in j^{th} cell, *i.e.* $W_{ij} = 0{:}\ Y_{ij} = 0$; $W_{ij} = 1{:}\ Y_{ij} > 0$.

Zero Inflated Negative Binomial Model

For any $\pi_{ij} \in [0, 1]$, Y_{ij} is assumed to follow a ZINB distribution [4,7,8]. The PMF of the ZINB distribution is expressed as follows.

$$f_{ZINB}(y) = P[Y_{ij} = y] = \pi_{ij}\delta_0(y) + (1 - \pi_{ij})f_{NB}(y) \qquad \forall\ y = 0, 1, 2, \dots \tag{1}$$

where, $f_{NB}(.)$: PMF of NB distribution; $\delta_0(.)$: Dirac's delta function. Here, $\delta_0(.)$ used to model the excess zeros in scRNA-seq data, and its PMF expressed as:

$$\delta_0(Y_{ij} = y) := \begin{cases} 1; & y = 0 \\ 0; & y \neq 0 \end{cases} \tag{2}$$

Now, the PMF of the ZINB distribution to model the UMI counts is given as:

$$P[Y_{ij} = y] = \begin{cases} \pi_{ij} + (1 - \pi_{ij})\left(\frac{\theta_{ij}}{\theta_{ij}+\mu_{ij}}\right)^{\theta_{ijk}} & when\ y = 0 \\ (1 - \pi_{ijk})\frac{G(y+\theta_{ij})}{G(y+1)G(\theta_{ij})}\left(\frac{\theta_{ij}}{\theta_{ij}+\mu_{ij}}\right)^{\theta_{ijk}}\left(\frac{\mu_{ij}}{\theta_{ij}+\mu_{ij}}\right)^{y}; & y > 0 \end{cases} \tag{3}$$

For, $Y_{ij} \sim ZINB(\pi_{ij}, \mu_{ij}, \theta_{ij})$, the expected value and variance of Y_{ij} can be obtained as (Supp. Document S1):

$$E(Y_{ij}) = (1 - \pi_{ij})\mu_{ij} \tag{4}$$

$$V(Y_{ij}) = (1 - \pi_{ij})\mu_{ij}\left(1 + \pi_{ij}\mu_{ij} + \frac{\mu_{ij}}{\theta_{ij}}\right) \quad (5)$$

If $\pi_{ij} = 0 \dot{\Rightarrow} ZINB(\pi_{ij}, \mu_{ij}, \theta_{ij}) \rightarrow NB(\mu_{ij}, \theta_{ij})$

If $\varphi_{ij} \rightarrow 0\ (No\ dispersion) \dot{\Rightarrow}\ ZINB(\pi_{ij}, \mu_{ij}, \theta_{ij})\ \rightarrow ZIP(\pi_{ij}, \mu_{ij})$

DEsingle

DEsingle [7] is a Zero Inflated Model (ZIM) based approach that employs the ZINB model (Eq. 3) to discriminate the observed zero values into two parts: dropout and true zeros (*i.e.,* from NB distribution). Under this model formulation, DEsingle is designed to overcome the issues of the excessive zeros observed in the scRNA-seq data. To detect DE genes between two cell groups, DEsingle first calculates the MLE of two ZINB populations parameters in Eq. 3, and then detects the DE genes using the LRT statistic through the constrained MLE of the two models' parameters under the null hypothesis. Here, the *p-values* for the genes were computed through executing the *DEsingle* function implemented in DEsingle R package [7].

DECENT

DECENT [8] is based on ZIM, precisely use the ZINB model given in Eq. 3 for fitting scRNA-seq count data, which also explicitly and accurately models the molecular capture process using a Beta-Binomial model. Here, the unobserved true UMI counts, Z_{ij}, are assumed to follow ZINB model (Eq. 3). Further, DECENT assumes the following models for different processes.

$$Z_{ij}; \pi_{ij}, s_j, \mu_{ij}, \theta_{ij} \sim ZINB(\pi_{ij}, s_j\mu_{ij}, \theta_{ij}) \quad (6)$$

$$Y_{ij} | Z_{ij} = k; p_{ij} \sim B(k, p_{ij}) \quad (7)$$

$$p_{ij} \sim Beta(a_{ij}, b_{ij}) \quad (8)$$

where, p_{ij} is the transcriptional capture rate for i^{th} gene of jth cell, *B*(.): Binomial distribution, $a_{ij}, and\ b_{ij}$ in Eq. 14 are the parameters of the beta distribution. DECENT uses the Expected Conditional Maximization (ECM) algorithm to calculate MLE of the ZINB model parameters (Eq. 6-8) using the

observed data through integrating molecular capturing procedure in the presence of external RNA-spike ins. To detect DE genes, DECENT uses the GLM framework in Eq. 15 to model the μ_{ij}.

$$log\, \mu_{ij} = \beta_{0i} + \beta_{1i} X_j + \tau_i' U_j \quad (9)$$

where, β_{0i}, β_{1i}, X_j has the usual meaning as in Eq. 6 and τ_i: regression coefficient of ith gene for j^{th} cell-level auxiliary U_j. The *p-values* for each gene are computed through LRT statistic under the GLM (Eq. 15), which is executed through *decent* function implemented in DECENT R package [8].

BPSC

BPSC [20] is an analytical method based on Beta-Poisson (BP) mixture model, designed to capture the distributional features of the scRNA-seq data, *i.e.*, non-integer expression or low expression values. In BPSC, the normalized data (Supp. Document S13), such as FPKM or, CPM, are modeled by using a four parameters BP model given in Eq. 16.

$$BP_4\left(Y_{ij} \middle| \alpha, \beta, \vartheta_1, \vartheta_2\right) = \vartheta_2 P\left(Y_{ij} \middle| \vartheta_1 Beta(\alpha, \beta)\right) \quad (10)$$

where, Y_{ij}: normalized value of the read counts; *P*(.): Poisson PMF; $\alpha, \beta, \vartheta_1, \vartheta_2$ are the parameters of the BP model. The expected value and variance of Y_{ij} is expressed as:

$$E\left(Y_{ij}\right) = \mu_{ij} = \vartheta_1 \vartheta_2 \frac{\alpha}{\alpha+\beta} \quad (11)$$

$$V\left(Y_{ij}\right) = \mu_{ij}\vartheta_2 + {\mu_{ij}}^2 \frac{\beta}{\alpha(\alpha+\beta+1)} \quad (12)$$

The MLEs of the parameters in Eq. 16 are estimated using the iterative weighted least-squares algorithm [20]. The DE analysis of the genes was carried out under the GLM framework given in Eq. 6. Further, *p-values* for the genes are computed through the LRT statistic by executing *BPglm* function implemented in the BPSC R package [20].

scDD

scDD [21] method, based on Logistic- Dirichlet mixture model, is designed to model the scRNA-seq data under a Bayesian modeling framework. It models the excess zeros in scRNA-seq data using logistic regression and models the non-zero counts using the conjugate Dirichlet model of normal distributions.

Here, the UMI counts are transformed to CPM measures through *cpm* function implemented in edgeR R package [22] followed by log-transformation. scDD uses a Bayesian modeling approach to detect DE genes between the two cellular groups. For this purpose, it computes an approximate Bayes factor score that compares the probability of DE with the probability of non-DE for each gene. The empirical gene *p-values* for the DE tests are computed using a permutation method. To execute this method, we used *scDD* function implemented in scDD R package [21].

MAST

MAST [23] uses a hurdle model approach for DE analysis and assumes conditional independence between expression rate (W_{ij}) and expression levels $\left(Y_{ij}\right)$ for each gene. It fits a logistic regression for W_{ij} and fits a Gaussian linear model for the continuous variable (Y_{ij} | W_{ij} = 1), which can be summarized as:

$$logit\left[\Pr\left(W_{ij} = 1\right)\right] = \boldsymbol{X_j \beta_i} \quad (13)$$

$$Pr(Y_{ij} = y \middle| W_{ij} = 1\Big) = N(\boldsymbol{X_j \beta_i}, \sigma_i^2) \quad (14)$$

In order to improve the inference for genes with sparse expression, the model parameters are fitted using an empirical Bayesian framework [23]. Finally, DE testing for genes is performed across the two cellular groups through the LRT statistic(s). For this purpose, we executed *zlm*, and *summary* functions for hurdle model fitting and DE analysis respectively implemented in MAST R package [23].

Monocle

Monocle [24,25] (updated as Monocle2 [25]), is a specially designed method for DE analysis, *i.e.* identifying DE genes that vary across different cell types or pseudo-times in scRNA-seq data. It uses a generalized additive model (GAMs) to model μ_{ij} under the GLM framework, *i.e.,* relating μ_{ij} to one or more predictors through GAMs for each gene and is expressed as:

$$log\mu_{ij} = \beta_{0i} + f_1(x_1) + f_2(x_2) + \cdots + f_M(x_M) \quad (15)$$

where, β_{0i}: regression co-efficient; x_j: predictor variable that represents group memberships of the cells; $f_j(.)$: smoothing functions, *e.g.*, cubic splines. Specifically, Y_{ij} across the cells are modeled using a Tobit model (approximately); thus, Monocle's GAM becomes:

$$\mu_{ij} = s\left(\delta_t\left(b_x, f_j\right)\right) + \varepsilon \tag{16}$$

where, $\delta_t\left(b_x, f_j\right)$: pseudo-time or cell type of a cell; f_j: cubic smoothing function (with three effective degrees of freedom), and ε: error term, follow a standard normal distribution. Further, Monocle performs DE testing of genes across cell groups through LRT statistic(s) by comparing full GLM with additional effects to a reduced GLM based on the NB model. For this purpose, *differentialGeneTest* function implemented in monocle R package [25] was executed.

EMDomics

EMDomics [26] is a general-purpose non-parametric method based on Earth Mover's Distance (EMD), developed for DE analysis of genomics data, *i.e.*, testing the gene's mean expressions difference between two cell groups significantly different from zero.

Let, $P_i = \{(p_{i1}, w_{p1}), (p_{i2}, w_{p2}) \dots, (p_{iM_1}, w_{pM_1})\}$ and $Q_i = \{(q_{i1}, w_{q1}), (q_{i2}, w_{q2}) \dots, (q_{iM_2}, w_{pM_2})\}$ be the signatures of ith gene across two cell groups; p_{im} ($m = 1, 2, \dots, M_1$) and q_{in} ($n = 1, 2, \dots, M_2$) are the centers of mth and n^{th} histogram in two cell groups; w_{pm} and w_{qn} are weights for m^{th} and n^{th} cell in two groups. The EMD score for i^{th} gene is computed through Eq. 27.

$$EMD_i = \frac{\sum_{m=1}^{M_1} \sum_{n=1}^{M_2} f_{mn}^i d_{mn}^i}{\sum_{m=1}^{M_1} \sum_{n=1}^{M_2} f_{mn}^i} \tag{17}$$

where, d_{mn}^i: Euclidean distance between m^{th} and n^{th} cell across two groups for i^{th} gene and f_{mn}^i: coefficient of flow from m^{th} to n^{th} cell for i^{th} gene and determined through minimizing the cost function in Eq. 28.

$$Cost^i(P, Q, F) = \sum_{m=1}^{M_1} \sum_{n=1}^{M_2} f_{mn}^i d_{mn}^i \tag{18}$$

Here, the EMD test statistic reflects the overall difference between two normalized distributions (for two cell groups), usually assessed through statistical significance using permutation test. For this purpose, *calculate_emd* function implemented in EMDomics R package [26] was executed.

NODES

NODES [27] is a non-parametric method used for detecting DE genes across two cell groups through using normalized scRNA-seq data. Here, normalization is done through the Pseudo-Counted Quantile Normalization method [27]. The test statistic for *i^th^* gene (d_i) is given in Eq. 29.

$$d_i = \frac{|\bar{y}_{i1} - \bar{y}_{i2}|}{a_0 + \sigma_i} \quad (19)$$

where, a_0: computed as a fixed percentile (*e.g.*, 50th) of the standard errors $\{\sigma_i; i = 1,2,\ldots,N\}$, and $\bar{y}_{i1}, \bar{y}_{i2}$, and σ_i are defined in Eq. 25. The *p-values* for the genes are computed through the permutation test through executing the *NODES* function implemented in NODES R package [27].

2. Document S2: Count data models

2.1 *Negative Binomial Distribution*

Most of the popular Differential Expression (DE) analysis tools, *e.g.* DESeq [1], DESeq2 [2], edgeR [3], *etc*., for bulk RNA-sequencing (RNA-seq) study assume the RNA-seq read counts to follow a Negative Binomial (NB) distribution, and subsequently, DE analysis is performed under Generalized Linear Model (GLM) framework.

Let, Y_{ij}: random variable (*rv*) representing the RNA-seq read counts of ith (i = 1, 2, …, N) gene of jth (j = 1, 2, …, M) cell; μ_{ij}: mean of ith gene of jth cell in i^{th} cell; θ_{ij} ($= \varphi_{ij}^{-1}$) and φ_{ij}: size and dispersion parameters respectively of ith gene in j^{th} cell for NB distribution.

Further, the Probability Mass Function (PMF) of the NB distribution is expressed as:

$$f_{NB}(y) = P[Y_{ij} = y] = \frac{G(y + \theta_{ij})}{G(y+1)G(\theta_{ij})} \left(\frac{\theta_{ij}}{\theta_{ij} + \mu_{ij}}\right)^{\theta_{ij}} \left(\frac{\mu_{ij}}{\theta_{ij} + \mu_{ij}}\right)^{y} \;\; \forall\, y = 0, 1, 2, \ldots \quad (20)$$

where, $\mu_{ij} \geq 0;\ \theta_{ij} > 0$ are the parameters of NB distribution, $G(.)$: Gamma function. Then, the expected value and variance of Y_{ij} is shown as:

$$E(Y_{ij}) = \mu_{ij} \tag{21}$$

$$V(Y_{ij}) = \mu_{ij} + \frac{{\mu_{ij}}^2}{\theta_{ij}} = \mu_{ij} + \varphi_{ij} \tag{22}$$

If $\varphi_{ij} \to 0\ (No\ dispersion)\ \dot{\Rightarrow} NB(\mu_{ij}, \theta_{ij})\ \to Poisson(\mu_{ij})$

2.2 *Zero Inflated Negative Binomial Model*

The proportions of zeros in single cell RNA-sequencing (scRNA-seq) data are higher as compared to bulk RNA-seq data due to low efficiency of mRNA capture efficiency, lower abundance of transcriptomics in single cell, amplification bias, *etc*. Therefore, the application of NB based bulk RNA-seq DE tools leads to several technical problems including lower statistical power to detect DE genes in scRNA-seq studies [4,5]. So, specialized scRNA-seq DE tools, *e.g.* ZINB-Wave [6], DEsingle [7], DECENT [8], etc. are developed based on the assumption that the observed scRNA-seq read counts follow a Zero Inflated Negative Binomial (ZINB) Distribution.

Let, Y_{ij}: *rv* representing the read (UMI) counts in scRNA-seq data of ith (i = 1, 2, …, N) gene of jth (j = 1, 2, …, M) cell; μ_{ij}: mean of ith gene of jth cell in i^{th} cell; θ_{ij} $(= \varphi_{ij}^{-1})$ and φ_{ij}: size and dispersion parameters respectively of ith gene in j^{th} cell for NB distribution; π_{ij}: zero inflation (*i.e.* the probability for a count to be an excess zero in a cell) parameter for i^{th} gene of jth cell.

For any $\pi_{ij} \in [0, 1]$, Y_{ij} is assumed to follow a ZINB distribution [4,7,8]. The PMF of the ZINB Distribution expressed as follows.

$$f_{ZINB}(y) = P[Y_{ij} = y] = \pi_{ij}\delta_0(y) + (1 - \pi_{ij})f_{NB}(y) \quad \forall\, y = 0, 1, 2, \ldots \tag{23}$$

where, $f_{NB}(.)$: PMF of NB distribution (Eq. 1); $\delta_0(.)$: Dirac's delta function. Here, $\delta_0(.)$ used to model the excess zeros in the data, and its PMF is expressed as:

$$\delta_0\left(Y_{ij} = y\right) := \begin{cases} 1; & y = 0 \\ 0; & y \neq 0 \end{cases} \tag{24}$$

Now, the PMF of the ZINB distribution to model the read counts from scRNA-seq data is given in Eq. 6.

$$P\left[Y_{ij} = y\right] = \begin{cases} \pi_{ij} + \left(1 - \pi_{ij}\right)\left(\frac{\theta_{ij}}{\theta_{ij}+\mu_{ij}}\right)^{\theta_{ijk}} & when\ y = 0 \\ \left(1 - \pi_{ijk}\right)\frac{G\left(y + \theta_{ij}\right)}{G(y+1)G\left(\theta_{ij}\right)}\left(\frac{\theta_{ij}}{\theta_{ij}+\mu_{ij}}\right)^{\theta_{ijk}}\left(\frac{\mu_{ij}}{\theta_{ij}+\mu_{ij}}\right)^{y}; & y > 0 \end{cases} \tag{25}$$

Now, $Y_{ij} \sim ZINB\left(\pi_{ij}, \mu_{ij}, \theta_{ij}\right)$, then the expected value and variance of Y_{ij} can be obtained as follows:

$$E\left(Y_{ij}\right) = \left(1 - \pi_{ij}\right)\mu_{ij} \tag{26}$$

$$V\left(Y_{ij}\right) = \left(1 - \pi_{ij}\right)\mu_{ij}\left(1 + \pi_{ij}\mu_{ij} + \frac{\mu_{ij}}{\theta_{ij}}\right) \tag{27}$$

If $\pi_{ij} = 0 \Rightarrow ZINB\left(\pi_{ij}, \mu_{ij}, \theta_{ij}\right) \rightarrow NB\left(\mu_{ij}, \theta_{ij}\right)$

If $\varphi_{ij} \rightarrow 0\ (No\ dispersion) \Rightarrow ZINB\left(\pi_{ij}, \mu_{ij}, \theta_{ij}\right) \rightarrow ZIP\left(\pi_{ij}, \mu_{ij}\right)$

2.3 *Poisson Distribution*

Poisson Distribution (PD) are also extensively used for analysis of count data obtained from bulk RNA-seq or scRNA-seq experiments. The PMF of PD can be expressed as:

$$f_{PD}(y) = P\left[Y_{ij} = y\right] = \frac{e^{-\mu_{ij}}\mu_{ij}{}^{y}}{G(y+1)}\ \forall\ y = 0, 1, 2, \ldots \tag{28}$$

$$E\left(Y_{ij}\right) = Var\left(Y_{ij}\right) = \mu_{ij} \tag{29}$$

2.4 *Zero Inflated Poisson Distribution*

Poisson model has very strict assumptions, *i.e.*, mean equals the variance, which is often violated in scRNA-seq data analysis. When the variance is too large because there are many 0s as well as a few very high values for expression counts [9]. In this case, a better solution is often the ZIPD model.

The PMF of ZIPD distribution can be expressed as:

$$f_{ZIPD}(y) = P\left[Y_{ij} = y\right] = \pi_{ij} I(y = 0) + \left(1 - \pi_{ij}\right) f_{PD}(y) \qquad \forall\, y = 0, 1, 2, \ldots \tag{30}$$

$$= \begin{cases} \pi_{ij} + \left(1 - \pi_{ij}\right) e^{\mu_{ij}} & when\, y = 0 \\ \left(1 - \pi_{ij}\right) \frac{e^{-\mu_{ij}} {\mu_{ij}}^{y}}{G(y+1)};\ \ y > 0 & \end{cases} \tag{31}$$

The mean and variance of ZIPD model is shown in Eq. 13 and 14, respectively.

$$E(Y) = \left(1 - \pi_{ij}\right)\mu_{ij} \tag{32}$$

$$Var(Y) = \left(1 - \pi_{ij}\right)\mu_{ij}\left(1 + \pi_{ij}\mu_{ij}\right) \tag{33}$$

2.5 *Hermite Distribution*

Hermite Distribution (HD) can be used to model the counts data [10]. Further, the PMF of HD is given in Eq. 15.

$$f_{HD}\left(Y_{ij} = y \middle| \alpha_{ij}, \beta_{ij}\right) = e^{-(\alpha_{ij} + \beta_{ij})} \sum_{k=0}^{[\frac{y}{2}]} \frac{{\alpha_{ij}}^{y-2k} {\beta_{ij}}^{k}}{G(y-2k+1)G(k+1)}\ \forall\, y = 0, 1, 2, \ldots \tag{34}$$

Further, the mean, variance, and dispersion index (*i.e.*, ratio between variance and mean) of rv $Y_{ij} \sim$HD (α, β) is given in Eq. 16 – 18.

$$E\left(Y_{ij}\right) = f\left(\alpha_{ij}, \beta_{ij}\right) = \left(\alpha_{ij} + 2\beta_{ij}\right) \tag{35}$$

$$Var\left(Y_{ij}\right) = \left(\alpha_{ij} + 4\beta_{ij}\right) \tag{36}$$

$$\varphi = g\left(\alpha_{ij}, \beta_{ij}\right) = 1 + 2\beta_{ij}/\left(\alpha_{ij} + 2\beta_{ij}\right) \tag{37}$$

The good-ness of fit of the above count data models, shown in Eq. 1, 6, 9, 12 and 15, were assessed through Akaike Information (AIC) and Bayesian Information (BIC) Criteria. The formula for AIC and BIC is expressed in Eq. 19 and 20.

$$AIC_m = -2logL_m + 2P_m \tag{38}$$

$$BIC_m = -2logL_m + P_m log(M) \tag{39}$$

where, L_m: Likelihood function for m^{th} model; P_m: Number of parameters in m^{th} model; AIC_m and BIC_m: AIC and BIC values for m^{th} model; M: Total number of cells in the data.

3. <u>Document S3</u>: Testing for zero inflation parameters for genes in scRNA-seq data

Here, we assume that the UMI (read) counts of the genes from a scRNA-seq study are generated through a ZINB population model given in Eq. 4 and 6. In order to test the statistical significance of the zero inflation parameters of ith gene π_i of the ZINB model, we adopt the following Generalized Likelihood Ratio Test (GLRT) procedure. Here, for the testing purpose, we define the following null hypothesis.

$$H_0: \pi_i = 0 \; vs. \; H_1: \pi_i \neq 0$$

where, H_0: null hypothesis; H_1: alternate hypothesis. In other words, null hypothesis tells us that k^{th} gene is not zero inflated, and subsequently, the scRNA-seq data structure is same as RNA-seq data. Further, if we fail to reject H_0, then the RNA-seq DE tools can be used for DE analysis of scRNA-seq data with the expectation of satisfactory results.

The above-mentioned test, $H_0 \; vs. H_1$, can be tested through GLRT and the test statistic is given in Eq. 21.

$$-2ln\alpha = -2\{l(\boldsymbol{\Omega}_i = \widehat{\boldsymbol{\Omega}}_{i0};\, Y_{ij}) - l(\boldsymbol{\Omega}_i = \widehat{\boldsymbol{\Omega}}_i;\, Y_{ij})\} \tag{40}$$

where, $\widehat{\boldsymbol{\Omega}}_{i0}$: Maximum Likelihood Estimator (MLE) of $\boldsymbol{\Omega}_i$ for i^{th} gene under the constraint of H_0 and $\widehat{\boldsymbol{\Omega}}_i$: unconstrained MLE of $\boldsymbol{\Omega}_i$ for i^{th} gene, $\boldsymbol{\Omega}_i$: parametric space for i^{th} gene, *i.e.*, $\boldsymbol{\Omega}_i =$

$\{\mu_i, \theta_i, \pi_i\}$. The test statistic in Eq. 31 is asymptotically distributed as Chi-square distribution with 1 degree of freedom under H_0.

We applied the above procedure to Tung et al.'s scRNA-seq data to test the statistical significance of the zero inflation parameters of genes. The results are shown in Figure S1. It can be observed that most of the genes in scRNA-seq data is found to be zero inflated as their corresponding *p-values* are less than the level of significance value (Figure S1). This finding motivates us to develop a statistical approach for testing of differential zero inflation of genes.

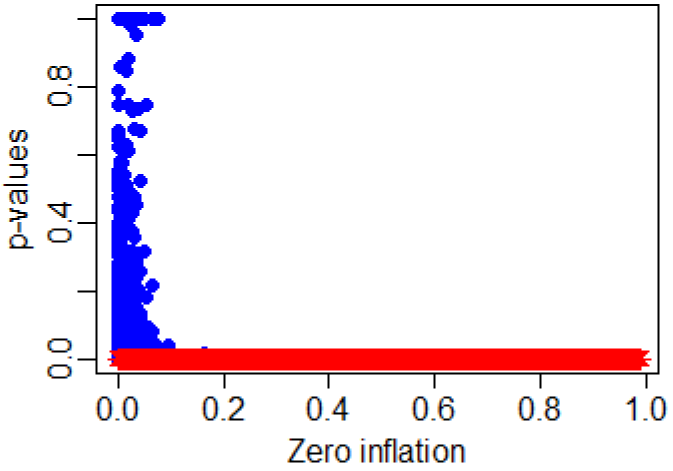

Figure S1. Plotting of estimated value of zero inflation parameter and their corresponding *p-values*. X-axis represents estimated values of zero inflation, (higher value of zero inflation parameter means a greater number of zeros found in the expression vector of that gene) and Y-axis represents the computed statistical significance value for the zero-inflation parameter, lesser the value represents the gene is more zero inflated.

4. <u>Document S4</u>: Statistical testing for overdispersion parameters in scRNA-seq data

For testing the statistical significance of the dispersion parameter of ith gene θ_i of the ZINB model, we adopt the following GLRT procedure. Here, for the testing purpose, we define the following null hypothesis.

$$H_0: \theta_i = 0 \ vs. \ H_1: \theta_i \neq 0$$

where, H_0: null hypothesis; H_1: alternate hypothesis. In other words, null hypothesis tells us that i^{th} gene is not dispersed, means the mean is same as the variance and subsequently, the scRNA-

seq count data is obtained from a Poisson model. Further, if we fail to reject H_0, then we can say UMI counts scRNA-seq data is not overdispersed and simply fitting a Poisson model will give satisfactory results.

The above-mentioned test, $H_0\ vs.H_1$, can be tested through GLRT and the test statistic is given in Eq. 22.

$$-2ln\alpha = -2\{l(\mathbf{\Omega}_i = \widehat{\mathbf{\Omega}}_{i0};\ Y_{ij}) - l(\mathbf{\Omega}_i = \widehat{\mathbf{\Omega}}_i;\ Y_{ij})\} \quad (41)$$

where, $\widehat{\mathbf{\Omega}}_{i0}$: MLE of $\mathbf{\Omega}_i$ for i^{th} gene under the constraint of H_0 and $\widehat{\mathbf{\Omega}}_i$: unconstrained MLE of $\mathbf{\Omega}_i$ for i^{th} gene, $\mathbf{\Omega}_i$: parametric space for i^{th} gene, *i.e.*, $\mathbf{\Omega}_i = \{\mu_i, \theta_i, \pi_i\}$. The test statistic in Eq. 22 is asymptotically distributed as Chi-square distribution with 1 degree of freedom under H_0.

We applied the above procedure to Tung et al.'s scRNA-seq data to test the statistical significance of the dispersion parameters of genes. The results are shown in Figure S2. It can be observed that all the genes in Tung's scRNA-seq data is found to be zero inflated as their corresponding *p-values* are less than the level of significance value (at alpha = 0.0001) (Figure S2). This finding is well reported in literature.

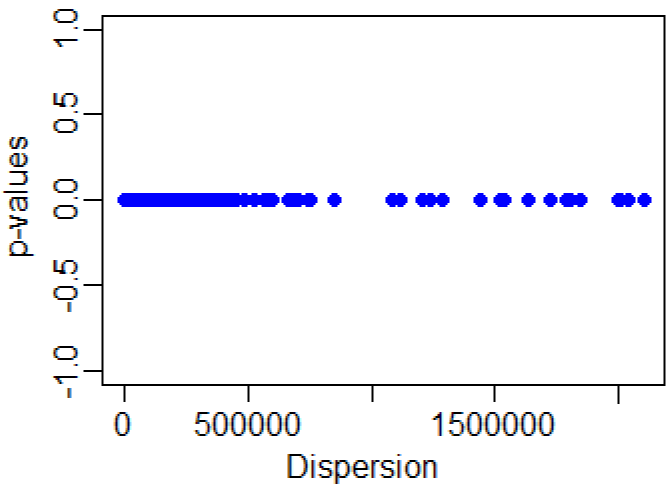

Figure S2. Testing for statistical significance of dispersion parameters. X-axis represents estimated values of overdispersion parameter through a ZINB model, and Y-axis represents the computed statistical significance value for the overdispersion parameter, lesser the value represents the gene is more overdispersed.

5. <u>Document S5</u>: Application of Count Data Models to Zero-Inflated and Overdispersed Real Datasets

In this section, we discuss about the fitting and suitability of different count data models such as NB, ZINB, PD, ZIPD and HD to the zero inflated and over dispersed datasets. These data set include, Embryonic Mouse Cysts count data and scRNA-seq UMI read counts data of a single gene, *i.e.* ENSG00000162585 from Tung's data [11].

4.1 *Embryonic Mouse Cysts Data*

Earlier experimental studies have shown that the scRNA-seq (UMI) read count data is zero inflated and over dispersed [12–18]. Hence, we consider a published zero-inflated and over-dispersed data on counts of cysts in embryonic mouse [19] to study the suitability of different discrete models. Here, we consider data on counts of cysts in embryonic mouse kidneys which had been subjected to steroids, taken from McElduff et al. [19]. This data reveals the details of the effect of a low protein diet in mice on kidney development in their offspring. Data on counts of cysts in embryonic mouse kidneys which had been subjected to steroid were featured in this study. Then, the count data models, such as NBD, ZINB, PD, ZIPD and HD are fitted on this data. Further, the parameters of these models are estimated through Maximum Likelihood Estimation (MLE) method. The observed frequencies and expected frequencies from different count models along with their estimated parameters are shown in Table S1. The goodness of fit of the above models to this experimental data is assessed through AIC and BIC.

Table S1. Fitting of well-known discrete models to over-dispersed and zero-inflated cyst count data.

Read	Obs. Freq.	Exp. Freq. NBD	Exp. Freq. ZINBD	Exp. Freq. PD	Exp. Freq. ZIPD	Exp. Freq. HD
0	65	63.29	64.99	25.1	65.03	45.36
1	14	17.56	14.01	37.32	5.1	13.75

2	10	8.98	9.11	27.74	8.87	28.92
3	6	5.72	6.27	13.74	10.28	8.35
4	4	3.91	4.44	5.11	8.93	9.19
5	2	2.79	3.2	1.52	6.21	2.53
6	2	2.04	2.33	0.38	3.6	1.94
7	2	1.52	1.71	0.08	1.79	0.51
8	1	1.15	1.26	0.01	0.78	0.31
9	1	0.88	0.93	0	0.3	0.08
10	1	0.68	0.69	0	0.1	0.04
11	2	0.52	0.52	0	0.03	0.01
12	1	0.41	0.38	0	0.01	0
Total	111	110.95	110.84	111	111.03	110.99
Parameters (MLE)		μ=1.49 θ=0.31	$\mu = 2.285$ $\theta = 0.698$ $\pi = 0.349$	$\mu = 1.486$	$\mu = 3.476$ $\pi = 0.572$	$\mu = 1.487$ $\varphi = 1.796$
#Parameters		2	3	1	2	2
Likelihood		-175.22	-172.8	-263.25	-191.9	-202.84
AIC		354.44	351.60	528.50	387.80	409.68
BIC		354.53	351.74	528.55	387.89	409.77

#Parameters: number of parameters; μ: Mean; θ: size; π: zero-inflation probability; φ: dispersion index (ratio of variance to mean); AIC: Akaike Information Criterion; BIC: Bayesian Information Criterion; Obs. Freq: Observed Frequency; Exp. Freq. NBD: computed expected frequency through NB model; Exp. Freq. ZINB: computed expected frequency through ZINB model; Exp. Freq. PD: computed expected frequency through Poisson model; Exp. Freq. ZIPD: computed expected frequency through ZIPD model; Exp. Freq. HD: computed expected frequency through HD model

It is observed that the expected frequencies computed from ZINB are closer to their observed values as compared to other models. Further, the AIC and BIC values for ZINB is lowest followed by NB model for the given zero inflated and over dispersed cyst count data as compared to PD, ZIPD and HD (Table S1). This indicates, for fitting over-dispersed and zero inflated datasets like scRNA-seq data, ZINB model provides a better fit as compared to other count models, *i.e.,* NB, PD, ZIPD and HD (Tables S1). Moreover, we validate the above claim by using another overdispersed and zero-inflated dataset from scRNA-seq study.

4.2 *Application to scRNA-seq read counts data*

Here, we fitted the considered count data models, such as NB, ZINB, PD, ZIPD and HD to the scRNA-seq read counts of ENSG00000162585 gene taken from Tung's data (available

at https://github.com/jdblischak/singleCellSeq). The observed and expected frequencies computed through different count models for each read sequences along with estimated values of the parameters are shown in Table S2.

Table S2. Fitting of well-known discrete models to over-dispersed and zero-inflated scRNA-seq read count data.

UMI Reads	Obs. Freq.	Pred. Freq. NB	Pred. Freq. PD	Pred. Freq. HD	Pred. Freq. ZINB	Pred. Freq. ZIP
0	115	108.05	0.09	4.82	126.82	115
1	84	57.92	0.78	3.34	56.96	0.06
2	45	42.58	3.37	20.33	39.79	0.36
3	33	34.13	9.73	13.54	31.11	1.34
4	31	28.48	21.03	42.8	25.61	3.73
5	18	24.34	36.39	27.48	21.73	8.32
6	12	21.13	52.46	59.94	18.79	15.44
7	10	18.53	64.83	37.17	16.48	24.56
8	7	16.39	70.11	62.84	14.6	34.19
9	9	14.59	67.38	37.71	13.03	42.31
10	4	13.05	58.29	52.62	11.7	47.12
11	6	11.72	45.84	30.59	10.56	47.71
12	12	10.56	33.04	36.67	9.58	44.28
13	4	9.54	21.99	20.68	8.71	37.94
14	3	8.64	13.59	21.87	7.95	30.18
15	5	7.84	7.84	11.98	7.27	22.41
16	8	7.13	4.24	11.39	6.67	15.6
17	6	6.5	2.16	6.07	6.13	10.22
18	4	5.93	1.04	5.27	5.64	6.32
19	7	5.41	0.47	2.74	5.2	3.71
20	5	4.95	0.2	2.19	4.8	2.06
21	5	4.53	0.08	1.11	4.43	1.09
22	4	4.15	0.03	0.83	4.1	0.55
23	5	3.8	0.01	0.41	3.8	0.27
24	6	3.49	0	0.29	3.53	0.12
25	4	3.21	0	0.14	3.27	0.06
26	7	2.95	0	0.09	3.04	0.02
27	5	2.71	0	0.04	2.83	0.01
28	5	2.49	0	0.03	2.63	0
29	3	2.29	0	0.01	2.45	0
30	2	2.11	0	0.01	2.28	0
31	2	1.95	0	0	2.12	0

33	5	1.65	0	0	1.85	0
34	5	1.53	0	0	1.72	0
35	7	1.41	0	0	1.61	0
36	3	1.3	0	0	1.5	0
39	4	1.03	0	0	1.23	0
40	2	0.95	0	0	1.15	0
41	1	0.88	0	0	1.08	0
42	3	0.81	0	0	1.01	0
43	1	0.75	0	0	0.94	0
46	3	0.59	0	0	0.78	0
47	1	0.55	0	0	0.73	0
49	2	0.47	0	0	0.64	0
50	2	0.44	0	0	0.6	0
Parameters (MLE)		μ=8.14 θ=0.574	μ=8.65	μ=8.651 φ=1.92	μ=8.652 θ=0.47377 π=1.173e-05	μ=11.1373 π=0.224

Parameters: parameters estimated through MLE; μ: Mean; θ: size; π: zero-inflation probability; φ: dispersion index (ratio of variance to mean); Obs. Freq: Observed Frequency; Pred. Freq. NBD: computed predicted frequency through NB model; Pred. Freq. ZINB: computed predicted frequency through ZINB model; Pred. Freq. PD: computed predicted frequency through Poisson model; Pred. Freq. ZIPD: computed predicted frequency through ZIPD model; Pred. Freq. HD: computed predicted frequency through HD model

It is observed that the expected frequencies computed from ZINB model are closer to their observed counter parts as compared to other models, such as NB, PD, ZIPD and HD (Table S2). Further, the fitting of the discrete models in terms of density plots are shown in Figure S3. This indicates, for fitting overdispersed and zero inflated scRNA-seq data, ZINB model provides a better fit to the observed scRNA-seq data as compared to other count models (Tables S2, Figure S3). Therefore, from the above applications of discrete models to zero-inflated and overdispersed scRNA-seq data, we can conclude that the ZINB model provides better fit to the data and better estimates of the parameters as compared to NB model. In other words, NB model is extensively used for modeling and fitting of bulk RNA-seq count data. But it performed poor when it is used for fitting the scRNA-seq data, which is simultaneously zero inflated and overdispersed (Figure S3). It can be observed that, for fitting overdispersed and zero inflated datasets like scRNA-seq data, ZINB model provides a better estimate of the mean and dispersion parameters as compared

to other count data models (Table S2, Figure S3). More specifically, we test the ability of NB, and ZINB models to estimate the mean and dispersion parameters for scRNA-seq count data through simulation, which is described in the following section.

References

[1] Love M I, Huber W and Anders S 2014 Moderated estimation of fold change and dispersion for RNA-seq data with DESeq2 *Genome Biol.*

[2] Malhotra A, Das S, Rai SN 2022 Analysis of Single-Cell RNA-Sequencing Data: A Step-by-Step Guide. *BioMedInformatics* 2, 43-61. https://doi.org/10.3390/biomedinformatics2010003

[3] Robinson M D, McCarthy D J and Smyth G K 2010 edgeR: a Bioconductor package for differential expression analysis of digital gene expression data *Bioinformatics* **26** 139–40

[4] Van den Berge K, Perraudeau F, Soneson C, Love M I, Risso D, Vert J-P, Robinson M D, Dudoit S and Clement L 2018 Observation weights unlock bulk RNA-seq tools for zero inflation and single-cell applications *Genome Biol.* **19** 24

[5] Risso D, Perraudeau F, Gribkova S, Dudoit S and Vert J-P 2018 A general and flexible method for signal extraction from single-cell RNA-seq data *Nat. Commun.* **9** 284

[6] Van den Berge K, Soneson C, Love M I, Robinson M D and Clement L 2017 zingeR: unlocking RNA-seq tools for zero-inflation and single cell applications *doi.org*

[7] Miao Z, Deng K, Wang X and Zhang X 2018 DEsingle for detecting three types of differential expression in single-cell RNA-seq data ed B Berger *Bioinformatics* **34** 3223–4

[8] Ye C, Speed T P and Salim A 2019 DECENT: differential expression with capture efficiency adjustmeNT for single-cell RNA-seq data ed B Berger *Bioinformatics* **35** 5155–62

[9] Zeileis A, Kleiber C and Jackman S 2008 Regression models for count data in R *J. Stat. Softw.*

[10] KEMP C D and KEMP A W 1965 Some properties of the “Hermite” distribution *Biometrika* **52** 381–94

[11] Tung P-Y, Blischak J D, Hsiao C J, Knowles D A, Burnett J E, Pritchard J K and Gilad Y 2017 Batch effects and the effective design of single-cell gene expression studies *Sci. Rep.* **7** 39921

[12] Islam S, Kjällquist U, Moliner A, Zajac P, Fan J B, Lönnerberg P and Linnarsson S 2011 Characterization of the single-cell transcriptional landscape by highly multiplex RNA-seq *Genome Res.*

[13] Boon W C, Petkovic-Duran K, Zhu Y, Manasseh R, Horne M K and Aumann T D 2011 Increasing cDNA yields from single-cell quantities of mRNA in standard laboratory reverse transcriptase reactions using acoustic microstreaming *J. Vis. Exp.*

[14] Macaulay I C and Voet T 2014 Single Cell Genomics: Advances and Future Perspectives *PLoS Genet.*

[15] Marinov G K, Williams B A, McCue K, Schroth G P, Gertz J, Myers R M and Wold B J 2014 From single-cell to cell-pool transcriptomes: Stochasticity in gene expression and RNA splicing *Genome Res.*

[16] Kharchenko P V., Silberstein L and Scadden D T 2014 Bayesian approach to single-cell differential expression analysis *Nat. Methods* **11** 740–2

[17] Pierson E and Yau C 2015 ZIFA: Dimensionality reduction for zero-inflated single-cell gene expression analysis *Genome Biol.*

[18] Wang Y and Navin N E 2015 Advances and Applications of Single-Cell Sequencing Technologies *Mol. Cell*

[19] McElduff F, Cortina-Borja M, Chan S K and Wade A 2010 When t-tests or Wilcoxon-Mann-Whitney tests won't do *Am. J. Physiol. - Adv. Physiol. Educ.*

[20] Vu T N, Wills Q F, Kalari K R, Niu N, Wang L, Rantalainen M and Pawitan Y 2016 Beta-Poisson model for single-cell RNA-seq data analyses *Bioinformatics*

[21] Korthauer K D, Chu L F, Newton M A, Li Y, Thomson J, Stewart R and Kendziorski C 2016 A statistical approach for identifying differential distributions in single-cell RNA-seq experiments *Genome Biol.*

[22] Robinson M D, McCarthy D J and Smyth G K 2010 edgeR: a Bioconductor package for differential expression analysis of digital gene expression data *Bioinformatics* **26** 139–40

[23] Finak G, McDavid A, Yajima M, Deng J, Gersuk V, Shalek A K, Slichter C K, Miller H W, McElrath M J, Prlic M, Linsley P S and Gottardo R 2015 MAST: a flexible statistical framework for assessing transcriptional changes and characterizing heterogeneity in single-cell RNA sequencing data *Genome Biol.* **16** 278

[24] Trapnell C, Cacchiarelli D, Grimsby J, Pokharel P, Li S, Morse M, Lennon N J, Livak K J, Mikkelsen T S and Rinn J L 2014 The dynamics and regulators of cell fate decisions are revealed by pseudotemporal ordering of single cells *Nat. Biotechnol.*

[25] Qiu X, Hill A, Packer J, Lin D, Ma Y-A and Trapnell C 2017 Single-cell mRNA quantification and differential analysis with Census *Nat. Methods* **14** 309–15

[26] Nabavi S, Schmolze D, Maitituoheti M, Malladi S and Beck A H 2016 EMDomics: A robust and powerful method for the identification of genes differentially expressed between heterogeneous classes *Bioinformatics*

[27] Sengupta D, Rayan N A, Lim M, Lim B and Prabhakar S 2016 *Fast, scalable and accurate differential expression analysis for single cells*

Table S3. Availability of the DE analytic approaches for scRNA-seq data analysis.

SN.	Methods	Vers.	Platform	Availability	Ref.
1	Seurat	4.0.4	R	https://cloud.r-project.org/package=Seurat	[80],

					[81]
2	SCDE	1.99.1	R,*	http://pklab.med.harvard.edu/scde/	[50]
3	scDD	1.16.0	R	https://www.bioconductor.org/packages/release/bioc/html/scDD.html	[58]
4	D3E	_	Python,*	https://github.com/hemberg-lab/D3E https://www.sanger.ac.uk/sanger/GeneRegulation_D3E	[51]
5	BPSC	0.99.2	R	https://github.com/nghiavtr/BPSC	[12]
6	MAST	1.19.0	R	https://github.com/RGLab/MAST	[53]
7	Monocle2	2.20.0	R	www.bioconductor.org/packages/release/bioc/html/monocle.html	[44], [45]
8	DEsingle	1.12.0	R	https://www.bioconductor.org/packages/release/bioc/html/DEsingle.html	[57]
9	DECENT	1.1.0	R	https://github.com/cz-ye/DECENT	[24]
10	DESCEND	1.0.0	R	https://github.com/jingshuw/descend	[28]
11	EMDomics	2.22.0	R	https://www.bioconductor.org/packages/release/bioc/html/EMDomics.html	[65]
12	Sincera	0.99.0	R*	https://research.cchmc.org/pbge/sincera.html https://github.com/xu-lab/SINCERA	[66]
13	ZIAQ	3.4.0	R	https://github.com/gefeizhang/ZIAQ	[42]
14	sigEMD	0.21.1	R	https://github.com/NabaviLab/SigEMD	[93]
15	TASC		Python	https://github.com/scrna-seq/TASC	[26]
16	ZINB-Wave	1.14.2	R	https://bioconductor.org/packages/zinbwave	[32]
17	SwarnSeq	0.1.0	R	https://github.com/sam-uofl/SwarnSeq	[13]
18	NODES	0.0.0.9010	R	https://goo.gl/Ndx07M	[60]
19	BASiCS	2.5.7	R	https://github.com/catavallejos/BASiCS	[25]
20	NBID	0.1.2	R	https://bitbucket.org/Wenan/nbid	[31]
21	tradeSeq	1.6.0	R	http://www.bioconductor.org/packages/release/bioc/html/tradeSeq.html https://github.com/statOmics/tradeSeq	[46]
22	SC2P	1.0.2	R	https://github.com/haowulab/SC2P	[52]
23	RandomHurdle	NA	…	…	[94]
24	NYMP	NA	Python, R	https://github.com/pachterlab/NYMP_2018	[59]
26	scDEA	1.0.0	R	https://github.com/Zhangxfccnu/scDEA	[83]
27	IDEAS	0.0.9000	R	https://github.com/Sun-lab/ideas	[100]
28	SIMCD	1.0.0	R	https://github.com/namini94/SimCD	[95]
29	zingeR	0.1.0	R	https://github.com/statOmics/zingeR	[33], [34]
30	ROSeq	1.4.0	R	http://www.bioconductor.org/packages/release/bioc/html/ROSeq.html https://github.com/krishan57gupta/ROSeq	[61]
31	DTWscore	1.0	R	https://github.com/xiaoxiaoxier/DTWscore	[96]
32	SAMstrt	0.99.0	R	https://github.com/shka/R-SAMstrt	[97]
33	t-test	4.3.0	R	https://stat.ethz.ch/R-manual/R-	[10]

				devel/library/stats/html/00Index.html	
34	Wilcox	4.3.0	R	https://stat.ethz.ch/R-manual/R-devel/library/stats/html/00Index.html	[10]
35	Tweedieverse	1.0	R	https://github.com/himelmallick/Tweedieverse	[35]
36	scMMST	NA	R	www.frontiersin.org/articles/10.3389/fgene.2021.616686/full#supplementary-material	[98]
37	TPMM	NA	R, C++	https://github.com/shilab2017/two_part_mixed_model	[99]

Vers.: Version; SN.: Serial Number
